# Effect of Pterostilbene, a Natural Derivative of Resveratrol, in the Treatment of Colorectal Cancer through Top1/Tdp1-Mediated DNA Repair Pathway

**DOI:** 10.3390/cancers13164002

**Published:** 2021-08-09

**Authors:** Yutian Zhang, Ying Li, Changcheng Sun, Xiang Chen, Luyao Han, Tingqiang Wang, Jinfeng Liu, Xijing Chen, Di Zhao

**Affiliations:** Clinical Pharmacokinetics Laboratory, School of Basic Medicine and Clinical Pharmacy, China Pharmaceutical University, Nanjing 211198, China; 1831090230@stu.cpu.edu.cn (Y.Z.); 3219091356@stu.cpu.edu.cn (Y.L.); 3219091357@stu.cpu.edu.cn (C.S.); 3220090966@stu.cpu.edu.cn (X.C.); 3220090967@stu.cpu.edu.cn (L.H.); 3320092045@stu.cpu.edu.cn (T.W.); jinfengliu@cpu.edu.cn (J.L.)

**Keywords:** pterostilbene, resveratrol, topoisomerase 1, tyrosyl-DNA phosphodiesterase 1, colorectal cancer

## Abstract

**Simple Summary:**

Tyrosyl-DNA phosphodiesterase 1 (Tdp1) repairs the stalled Topoisomerase 1 (Top1)-DNA covalent complex. It is conceivable that Tdp1 inhibitors could act synergistically with Top1 inhibitors to enhance the effect of Top1 poisons. This study identified pterostilbene (PTE) and resveratrol (RE) to suppress these two proteins by binding to their active center. PTE and RE could inhibit the proliferation of various colorectal cancer cells and decrease Top1 and Tdp1 contents and mRNA expression in wild-type, constructed *Tdp*1 overexpressing CL187, *Top*1- or *Tdp*1- silenced CL187 cell lines. PTE exhibited better antitumor activity and safety in subcutaneous CL187 transplantion model and orthotopic transplantation model. PTE had no significant inhibitory effect on non-tumor cell proliferation in vitro and would not induce damage to liver, kidney, and other major organs.

**Abstract:**

Topoisomerase 1 (Top1) inhibitor is an effective anticancer drug, but several factors limit its clinical application such as drug inactivation, tyrosyl-DNA phosphodiesterase 1 (Tdp1)-mediated tumor drug resistance, and its toxicity. Our previous study identified pterostilbene (PTE) and resveratrol (RE) to suppress these two proteins by binding to their active center. PTE and RE could inhibit the proliferation of various colorectal cancer cells, induce cell apoptosis, and make cell cycle stay in G2/M phase in vitro. PTE and RE could decrease Top1 and Tdp1 contents and mRNA expression in wild-type, constructed *Tdp*1 overexpressing CL187, *Top*1- or *Tdp*1- silenced CL187 cell lines. PTE exhibited excellent antitumor activity in subcutaneous CL187 transplantation model (TGI = 79.14 ± 2.85%, 200 mg/kg, i.p.) and orthotopic transplantation model (TGI = 76.57 ± 6.34%, 100 mg/kg, i.p.; TGI = 72.79 ± 4.06%, 500 mg/kg, i.g.) without significant toxicity. PTE had no significant inhibitory effect on non-tumor cell proliferation in vitro and would not induce damage to liver, kidney, and other major organs. Overall, PTE and RE can inhibit the activity of Top1 enzyme and inhibit the DNA damage repair pathway mediated by Top1/Tdp1, and can effectively inhibit colorectal cancer development with low toxicity, thus they have great potential to be developed into a new generation of anti-tumor drugs.

## 1. Introduction

Since Wang et al. discovered the first protein that can change the topology of DNA in 1971 [1,2,3], researchers have extensively studied this DNA topoisomerase and found that, unlike normal cells, DNA topoisomerase shows high levels of expression independent of other factors in tumor cells. Therefore, inhibiting the activity of DNA topoisomerase can prevent the rapid proliferation of tumor cells. Nowadays, Topoisomerase 1 (Top1) enzyme is an important target of anti-tumor drugs, and Top1 inhibitors are widely used in clinical practice, the main chemotherapeutic drugs targeting Top1 were camptothecin (CPT) and its derivatives. Camptothecin and its derivatives are commonly used to treat breast and ovarian cancer. Topotecan is commonly used to treat ovarian and lung cancer [4], and irinotecan (CPT-11) is used in combination with 5-fluorouracil to treat colorectal cancer [5]. Unfortunately, the safety of these compounds is compromised by either their own toxicity or the toxicity of their active metabolites. For example, CPT-11′s active metabolite SN38 has a strong toxicity in addition to its antitumor effect [6]. With increasing doses, these drugs’ side effects such as diarrhea and neutropenia become serious, even leading to treatment interruption. In addition, in terms of administration, these drugs also have some shortcomings. For example, some drugs have so low solubility that the intravenous dripping time has to be extended to achieve the maximum therapeutic effect [7,8,9,10,11]. At physiological pH, CPT is easily hydrolyzed into CPT carboxylate, seriously affecting its efficacy [12]. In addition, due to the presence of the tyrosyl-DNA phosphodiesterase 1 (Tdp1) enzyme in cells, the activity of Tdp1 is critical to the Top1-mediated DNA repair process. Tdp1 has been reported to repair the CPT-induced DNA damage, thus causing drug resistance in tumor cells. Considering it, major pharmaceutical companies and research institutions have been devoted to the development of new Top1 pathway inhibitors without the above-mentioned disadvantages [13]. At present, the development of Top1–Tdp1 dual inhibitors is underway [14,15,16,17,18,19,20].

In the early studies, we screened pterostilbene (PTE) and resveratrol (RE) with the dual inhibitory capacities of Top1 and Tdp1 through the combination of computer simulation screening and in vitro cytotoxicity tests. Resveratrol (3,4,5-trihydroxystilbene) is a non-flavonoid polyphenol compound and a stilbene derivative. It is mainly found in grapes and red wine. It has antioxidation, anti-inflammation, heart protection, and anti-tumor activity [21]. At present, a large number of preclinical studies have indicated that RE is a promising drug for cancer prevention and treatment [22]. However, RE is metabolized too fast in the body and its bioavailability is low, which limit its effective applications [23]. Pterostilbene [3,5-dimethoxy-4′-hydroxy-trans-stilbene] is a natural methylated derivatives of RE. PTE has been reported to be far superior to RE in metabolism and absorption [24,25,26,27]. A large number of in vivo and in vitro studies have confirmed that pterostilbene can inhibit tumor growth by changing cell cycle, inducing apoptosis, and inhibiting cancer cell metastasis [28,29,30,31,32], and that it exhibited good efficacy in treating bladder cancer, colon cancer, breast cancer, liver cancer, lung cancer, and other aspects [27,33,34,35]. It is expected to be a safe and low toxicity Top1-Tdp1 double inhibitor.

This study aims to investigate the effects of PTE and RE on DNA repair pathway mediated by Top1 and Tdp1, and to explore the potential of stilbene derivative as a safer and less toxic targeted antitumor drug. Further elaboration of their anti-tumor mechanism will lay the foundation for the development of stilbene derivative drugs.

## 2. Materials and Methods

### 2.1. Chemicals and Materials

**Figure 1:**
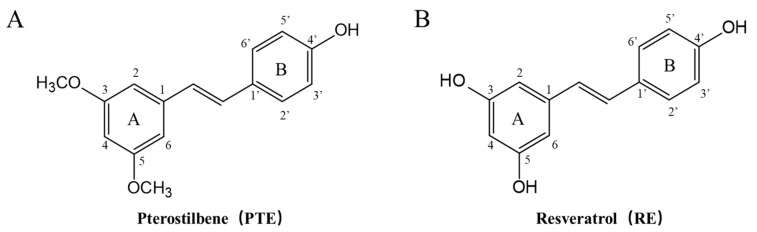
Structure of pterostilbene and resveratrol. (**A**) Pterostilbene (PTE). (**B**) Resveratrol (RE).

Pterostilbene (PTE, purity ≥ 99.0%, Figure 1A) was purchased from Dibai Biotechnology Co. (Shanghai, China), resveratrol (RE, purity ≥ 99.0%, Figure 1B), camptothecin (CPT, purity ≥ 97.0%) and irinotecan (CPT-11, purity ≥ 99.0%) were purchased from Macklin Biochemical Co. (Shanghai, China), Furamidine dihydrochloride (FM, purity ≥ 98.0%) was purchased from Sigma-Aldrich (St. Louis, MO, USA). Acetonitrile and methyl alcohol were HPLC-grade from Merck (Kenilworth, NJ, USA). Isopropanol, ethanol and dimethylsulphoxide (DMSO) were from Sinopharm Chemical Reagent Co., LTD (Shanghai, China). Ultrapure water obtained from a Milli-Q system (Millipore, Bedford, MA, USA).

### 2.2. Cell Lines and Cell Culture

#### 2.2.1. Cell Lines

Human colorectal cancer cell lines CL187, Colo205, HCT-8, SW480, Lovo, and HCT-116 were purchased from KeyGEN BioTECH Co. (Nanjing, China). 

#### 2.2.2. Construction and Transfection of *Tdp1* Overexpressed Plasmid

*Tdp1* overexpressed plasmid were purchased from TSINGKE Biological Technology (Shanghai, China), cloning vector was pcDNA3.1(+), cloning site was *BamHI—EcoRI*. Detailed plasmid information is presented in Supplementary Materials (Figure S1).

CL187 cells were cultured on 6-cm dishes at 37 °C for 24 h. The plasmid (2.5 μg) was diluted with 125 μL serum-free medium Opti-MEM, mixed gently, and incubated at room temperature for 5 min. Lipo 3000 (Invitrogen, Waltham, MA, USA) were diluted with 125 μL serum-free medium Opti-MEM, mixed and incubated for 5 min. Mixed the plasmid and Lipo 3000, incubated at room temperature for 20 min, the mixture was added to CL187 cells, the *Tdp*1 overexpressed plasmid were transfected into CL187 cells.

#### 2.2.3. RNA Interference/siRNA Transient Transfection

siRNA-*Top*1 and siRNA-*Tdp*1 were purchased from TSINGKE Biological Technology (Shanghai, China). siRNA-*Top*1 sequences (5′→3′) were: F: GGACAUAAGUGGAAAGAAGTT, R: CUUCUUUCCACUUAUGUCCTT. siRNA-*Tdp*1 sequences (5′-3′) were: F: CCAAAGAACUGAAAAUCAU, R: AUGAUUUUCAGUUCUUUGG. siRNA was transfected into the CL187 cells using the method described in Section 2.2.2.

#### 2.2.4. Cell Culture

CL187 were cultured in DMEM/F12 (KeyGEN BioTECH, Nanjing, China), HCT-8, Lovo and COLO205 were cultured in RPMI 1640 (Gibco, Carlsbad, CA, USA), SW480 were cultured in DMEM (KeyGEN BioTECH, Nanjing, China), HCT-116 were cultured in McCoy’s5A (Gibco, Carlsbad, CA, USA). All the culture media were supplemented with 10% FBS (Australia Origin; Gibco, Carlsbad, CA, USA), penicillin (100 U/mL) and streptomycin (100 μg/mL; KeyGEN BioTECH, Nanjing, China). The cells were incubated at 37 °C with 5% CO_2_. All cells were digested by trypsin (KeyGEN BioTECH, Nanjing, China) every two days.

### 2.3. Molecular Docking

The AutoDock 4.2.6 program (http://autodock.scripps.edu/news/autodock4-2.6 accessed on July 2019 to March 2021) was used for simulating molecular docking. This software was developed by the Scripps Research Institute and was freely available on the Internet. The structure of RE and PTE was prepared as a mol2 file using AutoDock Tools. The crystal structure of Top1 in complex with the poison CPT and covalent complex with a 22 base pair DNA duplex (PDB ID:1T8I), and that of Tdp1 in complex with vanadate, DNA, and a human topoisomerase 1-derived peptide (PDB ID:1NOP) were selected as the protein models. First, water molecules and solvent molecules were removed. Second, Gasteigere–Marsili charges and non-polar hydrogens were added. The other parameters for AutoDock were set as their defaults. Lamarckian genetic algorithm was chosen. Docking computation was run on a 64-bit computer. The docking results were visualized using the PyMOL program.

### 2.4. Anti-Tumor Efficacy of PTE and RE In Vitro

#### 2.4.1. Cell Viability Assay

PTE or RE were dissolved in DMSO and diluted with culture medium to 1–100 μM. The final concentration of DMSO was less than 0.1%, and 0.1% DMSO as negative control. CPT, as Top1 inhibitor, was diluted to 0.5 μM [36,37], and FM, as Tdp1 inhibitor was diluted to 25 μM [38]. Exponentially growing cells were seeded in 96-well plates (5 × 10^3^ cells/well) for 12 h. Various drugs were added separately and incubated for 24, 48, and 72 h. Cell viability was determined by CCK-8 (Beyotime Biotechnology, Shanghai, China) assay. Cell viability and inhibition rate were calculated with the following formulae:

Cell viability (%) = [OD_450_ (sample)/OD_450_ (control)] × 100 (1)

Inhibition rate (%) = 1 − cell viability (%)(2)

The drug concentration required for inhibiting cell proliferation by 50% (IC_50_) was determined by interpolation from dose-response curves.

#### 2.4.2. Apoptosis and Cell Cycle Analysis

Exponentially growing CL187 cells were seeded in 6-well plates (1.2 × 10^6^ cells/well) until a well was filled with cells. The RE or PTE at various concentrations were added separately into plate wells and incubated with cells for 48 h. The incubated cells were collected and treated by Annexin V-FITC/PI Apoptosis Detection Kit. The stained cells were analyzed with a flow cytometer (Becton-Dickinson, Franklin Lakes, NJ, USA), and data analyses were performed with BD Cell Quest^TM^ pro 6.0..

#### 2.4.3. Apoptosis Assessment by Hoechst 33,258 Staining

The clean cover glass was placed in a 6-well plate, and CL187 cells were inoculated. When the well was 50–80% full of cells, RE or PTE at various concentrations were added separately and incubated together with cells for 48 h to stimulate cell apoptosis. The supernatant was discarded. The cells were fixed, and cell apoptosis was determined with the Hoechst dyeing kit (Beyotime Biotechnology, Shanghai, China). Cell images were obtained with a Multimode microplate detection and cell imaging system (Cytation5, BioTek, Winusky, VT, USA). The apoptotic cells were characterized by intense fluorescence emitted by pyknotic fragmented nuclei. 

#### 2.4.4. Western Blot

CL187 cells were treated with RE or PTE for 6 or 12 h, collected, and placed into lysis buffer (KeyGEN BioTECH, Nanjing, China). The total protein content in the cell lysates was measured using a BCA kit. Equivalent amount of total protein (30 μg) was loaded onto 10% SDS-PAGE gel. Electrophoresis was performed at 80 V for 25 min. After the sample went through concentrated gel, the voltage was adjusted to 120 V. Electrophoresis was stopped when the bromophenol blue band approached the bottom of the gel. After being transferred to PVDF membranes, the samples were incubated separately with 3 types of primary antibodies Top1, Tdp1, and P53 rabbit mAb (CST, Boston, MA, USA), followed by the incubation with secondary antibody goat anti-rabbit IgG (HRP)-conjugated (1:3000). ECL kit (New Cell and Molecular Biotech, Suzhou, China) was used for color development, and the images were collected by the gel imaging system (Tanon-5200, Shanghai, China).

#### 2.4.5. RNA Isolation, Reverse Transcription and qPCR Analysis

CL187 cells were treated with different concentrations of RE and PTE for 6, 12, 24 h, collected, and added with TRIzol (Thermo Fisher Scientific, Waltham, MA, USA). RNA was precipitated by isopropyl alcohol and washed with 75% ethanol prepared from diethylpyrocarbonate (DEPC) water. The obtained total RNA was dissolved in DEPC water and reverse transcribed into cDNA by a Hifair Ⅲ 1st strand cDNA Synthesis SuperMix for qPCR Kit (Yeasen Biotech, Shanghai, China). The resultant cDNA was used as a template, and the primer sequences were (5′→3′): *Top*1: F: AGCATAAAGACAAACATAAAGAC, R: ATAATCAGCATCATCCTCATC; *Tdp*1: F: ACCAGAGTTCAGGAAGAAG, R: TCATAGAGCAGCAGCATC. *GAPDH*: F: CATTTCCTGGTATGACAACG, R: TCTTCCTCTTGTGCTCTTG. Transcription levels of *Top*1, *Tdp*1, and *GAPDH* genes were determined by qPCR. The qPCR was performed as follows: 95 °C for 10 min, 40 cycles of 95 °C for 10 s, and 60 °C for 30 s. The cycle threshold (Ct) value was determined, and the RQ value was calculated.

#### 2.4.6. Enzyme Active Analysis of Top1

##### Direct Enzyme Activity Assay

Different concentrations of the drug solution were prepared. The solvent was used as negative control group, and CPT (50 μM) was used as the positive control group. Each sample included 2 units of Top1 (TopoGEN, Buena Vista, CO, USA) and 250 ng super helical DNA (pHOT-1, TopoGEN, Buena Vista, CO, USA). The samples were incubated for 30 min at 37 °C, and then the reaction was stopped with SDS. After digestion with proteinase K (50 μg/mL, 60 min at 37 °C), samples were separated through electrophoresis (1% agarose gel). The electrophoresis was conducted at 180 V constant pressure for 20 min. The gel was immersed in the dye without EB for 30 min, and the images were collected by gel imaging system (Tanon-1600B, Shanghai, China).

##### Intracellular Enzyme Activity Assay

CL187 cells were treated with different concentrations of drugs for 6 h. The supernatant was discarded, and the cells were collected. The extracts of cytoplasm, mitochondria, and nucleus were obtained by the same method as described in Section 2.4.7. The protein concentration of each extract was detected by BCA kit. Enzyme activity reaction was carried out by using 125 ng super helical DNA (pHOT-1) and cell extract. The protein concentration of the extract was guaranteed to be the same in each group. The mixture solution of the pHOT-1 and the cell extract was added into the reaction buffer and incubated for 30 min at 37 °C. The reaction was terminated with loading buffer. The agarose gel electrophoresis was performed, and the images were collected. 

#### 2.4.7. Intracellular Drug Distribution

CL187 cells were inoculated in 6-well plates, until the well was full of the cells. Then the supernatant was discarded, and the cells were washed with PBS three times. The cells were added with the different concentrations of PTE or RE and incubated for 6 h. After supernatant was discarded, the cells were washed with cold Hank’s solution for three times to terminate cell metabolism. Cell cytoplasm, nucleus, and mitochondria were extracted with the nuclear and mitochondrial extraction kit (KeyGEN BioTECH, Nanjing, China). Cells were resuspended with 1.5 mL Lysis Buffer and transferred to a 2 mL glass homogenizer for grinding (0–4 °C ice bath). The homogenate was collected and centrifuged at 800× *g* at 4 °C for 5 min. The nuclei were distributed in the precipitate, while the mitochondria were distributed in the supernatant. The precipitate was resuspended with 0.5 mL Medium Buffer A. Another new precooled centrifuge tube was added with 1 mL Medium Buffer B, the suspension was placed on Medium Buffer B, centrifuged at 1000× *g* at 4 °C for 10 min, and the supernatant was discarded to obtain a relatively pure nuclear precipitation. The nuclei was resuspended with 50–100 μL Nuclear Store Buffer. A total of 0.5 mL of Medium Buffer C was added to another new precooled centrifuge tube, 0.5 mL of the supernatant of homogenate was carefully added along the wall of the tube, and covered the Medium Buffer C. The centrifugation was performed at 15,000× *g* at 4 °C for 10 min, the supernatant after centrifugation contained cytoplasmic components, and the mitochondria settled at the bottom of the tube. The mitochondrial precipitation was resuspended with 0.2 mL Wash Buffer, centrifuged at 15,000× *g* at 4 °C for 10 min, and the supernatant was discarded. A total of 50–100 μL of mitochondria Store Buffer was used to resuspend the mitochondrial precipitation. The protein concentration of each extract was detected by the BCA kit.

The drug contents of the extracted cell cytoplasm, nucleus, and mitochondria were determined by LC-MS/MS. The internal standard was Chlorzoxazone at 800 ng/mL. The UPLC system consisted of an autosampler SIL-30AC, pump LC-30AD, column oven CTO-30AC, and controller CBM-30A (Shimadzu, Kyoto, Japan). Chromatographic separations of RE and PTE were performed on the C18 column (150 × 2.0 mm, 2.1 µm, Shimadzu, VP-ODS). The binary mobile phase consisted of mobile Phase B (acetonitrile) and mobile Phase A (water with 5 mM ammonium acetate). The flow rate was set as 0.4 mL/min and the column temperature was maintained at 40 °C. The isocratic elution was 90% Phase B and 10% Phase A. The overall run time was 5.0 min. An ultra-high performance liquid chromatography (UPLC) system (LC-30A, Shimadzu, Kyoto, Japan) and a triple quadrupole tandem mass spectrometer (AB SCIEX 4000, AB SCIEX, Foster City, CA, USA) were combined to determine the contents of RE and PTE. Analyst 1.6.3 software (AB SCIEX, Framingham, MA, USA) was employed for data acquisition and analysis. For the determination of RE and PTE, the optimal instrument conditions were as follows: ion spray voltage of −4000 V, capillary temperature of 400 °C. Nitrogen was used as both the sheath and auxiliary gases with sheath gas of 30 Arb and auxiliary gas of 25 Arb. The collision energy (CE) for RE was −27 V, that for the PTE was −28 V, and that for the Chlorzoxazone was −20V. RE, PTE, and Chlorzoxazone were identified by electrospray negative ionization and multiple reaction monitoring (MRM) with the corresponding ion transitions of *m*/*z* 227.10→185.00, *m*/*z* 255.10→240.10, and *m*/*z* 168.00→132.10, respectively. The calibration range for the quantitation of each compound in all cellular components was 10 to 10,000 ng/mL. This bioanalytical method was validated according to the corresponding guides. 

### 2.5. Antitumor Efficacy of PTE In Vivo

#### 2.5.1. Subcutaneous CL187 Xenograft Model

The experimental procedures and the animal use and care protocols were approved by the Committee on Ethical Use of Animal Ethics Committee of Jiangsu KeyGEN BioTECH Co., LTD (Nanjing, China, Certificate No.: IACUC-006-1). BABLc/nude mice (18–20 g) 5 weeks old (half male and half female) were provided by Shanghai Slake Laboratory Animal Co., LTD. (Shanghai, China, SCXK 2017-0005,).

CL187 cell suspension was collected at 1 × 10^6^ /mL, and 0.1 mL cell was inoculated into the right inguinal region of nude mice. The tumor was no metastasis. The diameter of the transplanted tumor in nude mice was measured with a vernier caliper. After 16-day inoculation (the tumor grew to 90 mm^3^), the animals were randomly divided into 7 groups (6 mice per group). The drug was administered by intraperitoneal injection. Blank control group was injected with saline. Vector control group was injected with 5% DMSO and 95% saline containing 30% HP-β-CD. According to the preliminary test results. The experimental group was treated with PTE at concentrations of 25, 50, 100, and 200 mg/kg once a day. Positive control group was injected with irinotecan (20 mg/kg) twice a week [33,39], During the experiment, the changes of body weight of nude mice were recorded, and the anti-tumor effect of the samples was dynamically investigated by measuring tumor diameter. On Day 31 post treatment, the nude mice were sacrificed, and the tumor blocks were removed surgically, weighed, and recorded.

The 20 mg of the tumor was added into 1 mL RIPA lysis, and homogenized for 3 times under low temperature condition, and centrifuged at 12,000 rpm for 10 min. Subsequently, the supernatant was collected, and the contents of Top1 and Tdp1 were determined by WB as described in Section 2.4.4. The 20 mg of tumor was added into 1 mL Trizol, and homogenized for 3 times. The expression levels of *Top*1 and *Tdp*1 were analyzed using the method described in Section 2.4.5.

#### 2.5.2. Orthotopic CL187 Xenograft Model

The experimental procedures and the animal use and care protocols were approved by the Committee on Ethical Use of Animal Ethics Committee of Jiangsu KeyGEN BioTECH Co., LTD (Nanjing, China, Certificate No.: IACUC-001-18). BABLc/nude mice (18–20 g) 5 weeks old (half male and half female) were provided by Shanghai Lingchang Biotechnology Co., Ltd. (Shanghai, China, SCXK 2018-0003). 

The mice were anaesthetized with 10% chloral hydrate and fixed on the operating table. After the abdomen of the mice was disinfected with iodine, a small section of the large intestine was squeezed out of the surgical opening. The 0.1 mL (per mouse) CL187 cell suspension (1 × 10^7^ /mL) was inoculated into the colon wall of nude mice. After the inoculation, the large intestine was placed into the mouse abdominal cavity. After the wound was sutured, the mice were put into cages until they naturally woke up. After 10 days of inoculation, the animals were randomly divided into 4 groups (6 mice per group). Vector control group was intraperitoneally injected with 5% DMSO and 95% saline containing 30% HP-β-CD (i.p.). The drug dosage was designed according to the results of the previous pharmacokinetic experiment. Experimental Group 1 was intraperitoneal injected (i.p.) with PTE 100 mg/kg, and experiment Group 2 was intragastric administrated (i.g.) with PTE 500 mg/kg, once a day. The bioavailability of PTE was the same in the animals in the above regimens. Positive control group was injected with irinotecan, 20 mg/kg, twice a week. During the experiment, the changes of body weight of nude mice were recorded. After 21 days, the nude mice were sacrificed, the tumor blocks were removed surgically, weighed, and recorded.

#### 2.5.3. Histology and Immunohistochemistry Analyses

Tumor and organ tissues were paraffin-embedded and sectioned into 4 mm after being fixed in 4% of paraformaldehyde solution for 24 h. The sections were deparaffinized and rehydrated before detection. For histological observation, tumor sections were stained with hematoxylin and eosin (HE), and then observed by an inverted microscopy (Olympus, Japan).

For immunohistochemistry analysis, the sections were treated with 3% hydrogen peroxide for 10 min to block endogenous peroxidase, and then incubated with Ki67 primary antibody for overnight at 4 °C. Subsequently, the sections were treated with secondary antibody at room temperature for 20 min. After being washed with PBS, the sections were incubated with DAB chromogenic solution for 10 min, followed by rewashing in PBS. The sections were counterstained with hematoxylin after DAB coloration. The antibody specificity was confirmed by negative control without primary antibody treatment. Immunopositive areas were observed by an inverted microscope (Olympus, Japan).

### 2.6. Statistical Analysis

Statistical analysis was carried out by GraphPad Prism 6.0. The data were expressed as mean ± SD. Differences between groups were analyzed using the SPSS 20.0 one-way ANOVA analysis (SPSS, Inc.; Chicago, IL, USA). *p* < 0.05 or *p* < 0.01 were considered as significantly different or highly significantly different, respectively. 

## 3. Results

### 3.1. Antitumor Activity of PTE and RE In Vitro

#### 3.1.1. Cell Viability Assay

Antiproliferative activities of PTE and RE were tested against various human colorectal cells by CCK-8 assays with CPT and FM as the positive control. After the six experimental colon cancer cell lines were treated with RE or PTE for 24, 48, and 72 h, cell viabilities were significantly inhibited (Figure S2). The drug concentration required to IC50 values was determined by interpolation from dose-response curves (Table 1). PTE and RE inhibited the activities of various colorectal cancer cells in a dose-dependent manner in vitro. The higher the drug concentration was, the higher the inhibitory effect was. After 72 h treatment, the IC50 values of PTE and RE for six colon cancer cell lines (except SW480 cells) were lower than 30 μM. Compared with RE, PTE showed a stronger inhibitory effect on each colon cell lines. At the same concentration, the inhibitory effect of PTE on cell proliferation was better than that of FM, but was less good than that of CPT.

HCoEpiC-, HFL-1, and L02 are non-tumor cells that can proliferate in vitro. PTE and RE had no obvious inhibitory effect on the proliferation of these cells (Table S1, Figure S3). After 72 h drug treatment, the IC50 values of PTE for these three cell lines were 83.43, 130.2 and 148.1 μM, respectively, but the IC50 values of RE were all higher than 100 μM, indicating that PTE and RE had certain selectivity to tumor cells and non-tumor cells when they inhibited cell proliferation in vitro.

#### 3.1.2. Compounds Induce Apoptosis in CL187

**Figure 2:**
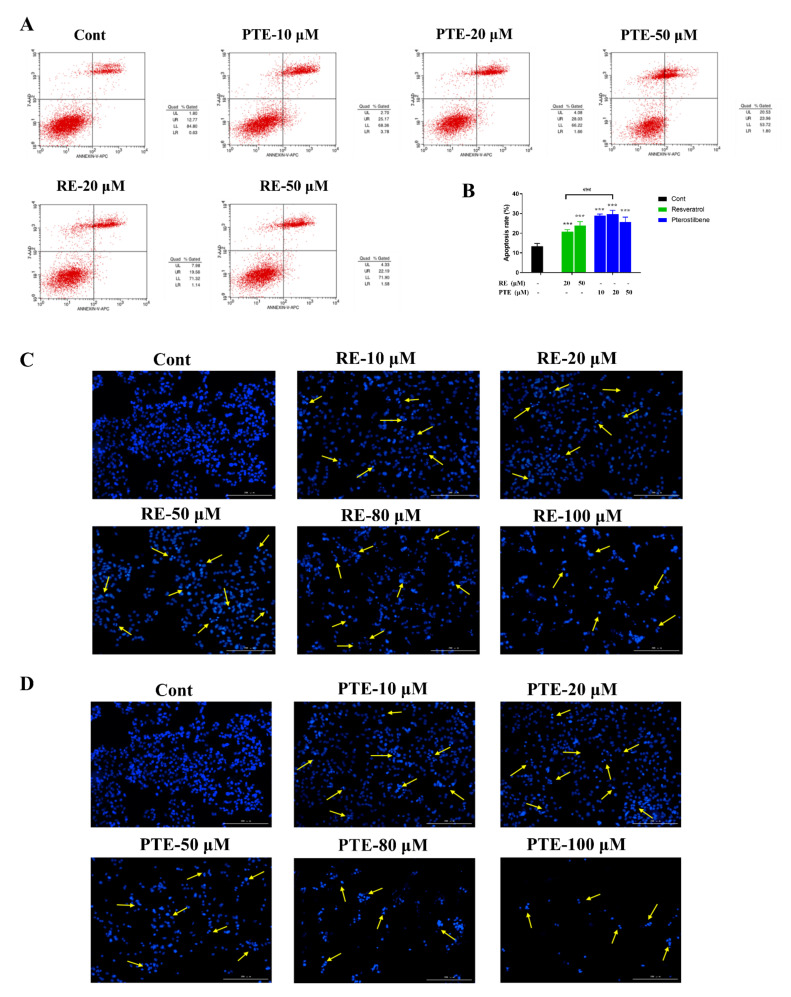
Apoptosis of CL187 cells treated with PTE and RE. (**A**) CL187 cells were treated with PTE or RE at different concentrations for 48 h, followed by flow cytometry. (**B**) Total apoptosis rate through flow cytometry. *** *p* < 0.001 vs. control. ns, not significant. Data are mean ± SD, *n* = 3. CL 187 cells were treated with RE (**C**) and PTE (**D**) at different concentrations for 48 h. Stained cells were observed under a multimode microplate detection and cell imaging system (×100). The cells with condensed chromatin and shrunken nuclei were defined as apoptotic cells, marked with yellow arrows in the picture.

Flow cytometry was used to detect the effect of PTE and RE on the apoptosis rate (the percentage of apoptotic cells, including early and late apoptotic cells) of CL187 cells (Figure 2A,B). Both PTE and RE induced CL187 cell apoptosis and increased the percentage of early and late apoptotic cells. The apoptosis rate in the PTE treatment group was 28.95%, 29.69% and 25.76% at PTE concentration of 10, 20, and 50 μM, and it was 20.70% and 23.77% in the RE treatment group at the concentration of 20 and 50 μM. The apoptosis inducing ability of PTE was significantly higher than that of RE. Especially, at lower concentration (10 μM), the apoptosis inducing ability of PTE was higher than that of RE at higher concentration (20 and 50 μM). In addition, in the PTE treatment group, the proportion of necrotic cells increased with the increase of drug concentration, indicating that PTE can not only induce the apoptosis of CL187 cells, but also promote the necrosis of some cells. The apoptotic cells were featured as pyknotic and fragmented nuclei. To observe nuclear shape of apoptotic cells, CL187 cells were treated with PTE and RE (at the concentrations of 10, 20, 50, 80, 100 μM) for 48 h, stained with Hoechst 33258, and were observed by living cell imaging system. Significant morphological changes were observed in nuclear chromatin. In the blank control group, the nuclei were stained into a less bright blue with homogeneous color. However, condensed chromatin was observed in the cells treated with RE or PTE and the apoptotic bodies were formed in the cells (Figure 2C, D). With the increasing drug concentration, the number of cells was gradually reduced, but the number of the shrinking nucleus was increased. At the same concentration, the number of cells in PTE treatment group was significantly smaller than that in RE treatment group, but the proportion of apoptotic cells was also significantly higher in PTE treatment group than in RE treatment group. 

**Figure 3:**
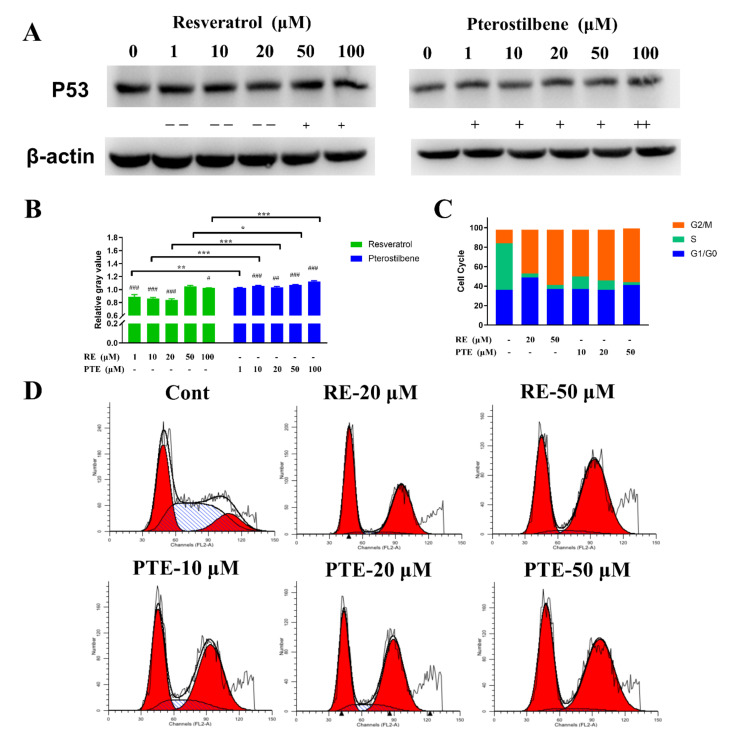
(**A**) WB probing for P53 in CL 187 cells treated with PTE and RE for 12 h. Loading was normalized based on the level of the internal control, β-actin. Compared with the control group, “+” or “−” was denoted when the change of gray value was between 0 and 10%, “++” or “− −” was denoted when the change of gray value was between 10–20%. (**B**) Gray value analysis of WB experiment results. * *p* < 0.05, ** *p* < 0.01, and *** *p* < 0.001 vs. RE treatment group. # *p* < 0.05, ## *p* < 0.01 and ### *p* < 0.001 vs. control group. ns, not significant. Data are mean ± SD, *n* = 3. (**C**) The percentage of cells that stay in different replication cycles. (**D**) RE and PTE arrested cell cycle at G2/M phase. CL 187 cells exposed to PTE or RE for 48 h were examined by flow cytometry assay.

PTE and RE promoted the expression of P53 (apoptosis-related protein) in CL187 cells in a dose-dependent manner (Figure 3A). The expression of P53 in the PTE group was significantly higher than that in the RE group (Figure 3B). This result was consistent with the above-mentioned observations of cell apoptosis detected by flow cytometry and Hoechst 33258 stain, indicating that PTE had a better ability to promote the apoptosis of CL187 cells than RE.

#### 3.1.3. Compounds Induce Cell Cycle Arrest in CL187

PTE and RE affected the cell cycle progression in CL187 cells (Figure 3D). The cell cycle changed significantly after RE or PTE treated cells for 48 h. The proportion of cells staying in S phase was decreased significantly, but the proportion of cells in G2/M phase was increased with the increase in drug concentration. The proportion of G2/M-phase cells in the RE treatment group (at concentration of 10 and 50 μM) were 45.34% and 57.76%, respectively, and that in the PTE treatment group (at concentration of 10, 20, and 50 μM) were 48.69%, 52.98% and 55.33%. The proportion of G2/M phase cells in the blank control group was 14.70%. These results indicated that PTE and RE may affect the cell replication cycle, promote the cell cycle to stay in the G2/M phase, thus blocking the DNA replication progress.

### 3.2. PTE and RE Inhibit the Enzyme Activity and Expression of Top1 and Tdp1 In Vitro

#### 3.2.1. Effect of PTE and RE on Top1 Enzyme Activity

Top1 enzyme activity assay was performed with a circular supercoiled plasmid DNA (pHOT1) as substrate. Top1 can relax this DNA at 37 °C in the presence of 10 mM MgCl_2_. However, when Top1 inhibitor blocks the enzyme activity of Top1, DNA will not be relaxed.

**Figure 4:**
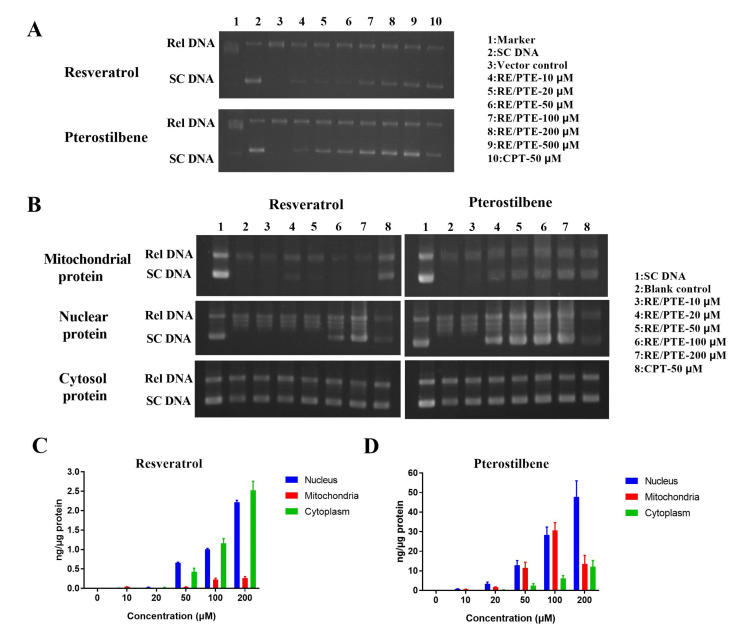
Effects of PTE and RE on Top1 relaxation activity. (**A**) Effects of PTE and RE at the different concentrations on Top1 relaxation activity. Lane 1, DNA marker; Lane 2, supercoiled plasmid DNA (pHOT-1, SC DNA); Lane 3, SC DNA+Top1; Lanes 4–9, SC DNA+Top1+RE/PTE; Lane 10, SC DNA+Top1+CPT. (**B**) Lane 1, SC DNA; Lane 2, SC DNA+ extract of CL 187 cells; Lanes 3–8, SC DNA+ extract of CL 187 cells treated with RE or PTE. Distribution of RE (**C**) and PTE (**D**) in CL 187 cells. Data are mean ± SD, *n* = 3.

After 30-min incubation of Top1 with RE or PTE, agarose gel electrophoresis was used for detecting DNA. As shown in Figure 4A, the sample in the Lane 2 was the supercoiled DNA (SC DNA) substrate. After gel electrophoresis, a small amount of relaxed DNA was found in the lane, indicating that a small amount of SC DNA was degraded during the reaction. The sample in Lane 3 was composed of SC DNA, Top1 enzyme, and reaction buffer. After the gel electrophoresis, the SC DNA was completely relaxed. The sample in Lane 10 consisted of SC DNA, Top1 enzyme, 50 uM CPT, and reaction buffer. After gel electrophoresis, both SC DNA and relaxing DNA were observed in Lane 10. These observations suggested that CPT could inhibit Top1 enzyme activity and inhibit the SC DNA relaxation process. The samples in Lanes 4 to 9 consisted of SC DNA, Top1 enzyme, different concentrations of PTE or RE, and reaction buffer. The gel electrophoresis results showed the increase in SC DNA and the decrease in relaxed DNA with the increase in PTE or RE concentration, indicating that both PTE and RE had the ability to inhibit Top1 enzyme activity in a dose-dependent manner.

The PTE at concentration of 20 uM, RE at the concentration of 100 uM and CPT at the concentration of 50 uM exhibited the same inhibitory effect, indicating that PTE had stronger inhibitory ability on Top1 enzyme activity than CPT and RE in vitro.

#### 3.2.2. Effect of PTE and RE on Top1 Enzyme Activity in CL187 

Top1 enzymes mainly exist in the nucleus and cell mitochondria, and there also exist a small amount in the cytoplasm. We found that PTE and RE inhibited the enzyme activity of Top1 in CL187 cells. After CL187 cells were treated with different concentrations of RE or PTE for 6 h, the total proteins in the nucleus, mitochondria, and cytoplasm were extracted. After the cell extract and SC DNA were incubated in the reaction buffer for 30 min, the amount of relaxed DNA was detected by agarose electrophoresis (Figure 4B). The sample in Lane 2 was used as a blank control group. In the extracted nucleus and mitochondria, Top1 enzyme activity was maintained, and SC DNA substrate was relaxed, but in the cytoplasm, there was almost no Top1 enzyme, thus the SC DNA was not relaxed. Considering this, the activity of Top1 enzyme in the cytoplasm would not be discussed below. CPT treatment group sample (at the concentration of 50 μM) in Lane 8 significantly inhibited the activity of Top1 in the nucleus and mitochondria of CL187 cells. The RE treatment group had a good inhibitory effect on the Top1 enzyme activity in cell mitochondria, but it had a poor inhibitory effect on the Top1 enzyme activity in the nucleus with a certain inhibitory effect observed only in the high concentration (100 and 200 μM) treatment groups. However, PTE treatment group significantly inhibited Top1 activity in the mitochondria and nucleus of cells. The inhibitory effect of PTE at the concentration of 20 μM on the intracellular Top1 enzyme activity was the same as that of CPT at the concentration of 50 μM.

Since the different inhibitory effects of PTE and RE on Top1 activity in cells were related to the different intracellular drug distributions, the drug distributions in the nucleus, mitochondria, and cytoplasm were detected after CL187 cells were incubated with RE or PTE for 6 h. As shown in Figure 4C, the concentration of RE was low in cells with its concentration of no more than 5.0 ng/μg. RE was widely distributed in the cytoplasm and nucleus, but there was relatively low RE concentration in the mitochondria. In contrast, the concentration of PTE was relatively high in the cell (Figure 4D) with the maximum concentration of PTE of more than 70.0 ng/μg. PTE concentration was relatively high in the nucleus and mitochondria, and relatively low in the cytoplasm.

#### 3.2.3. Molecular Docking

**Figure 5:**
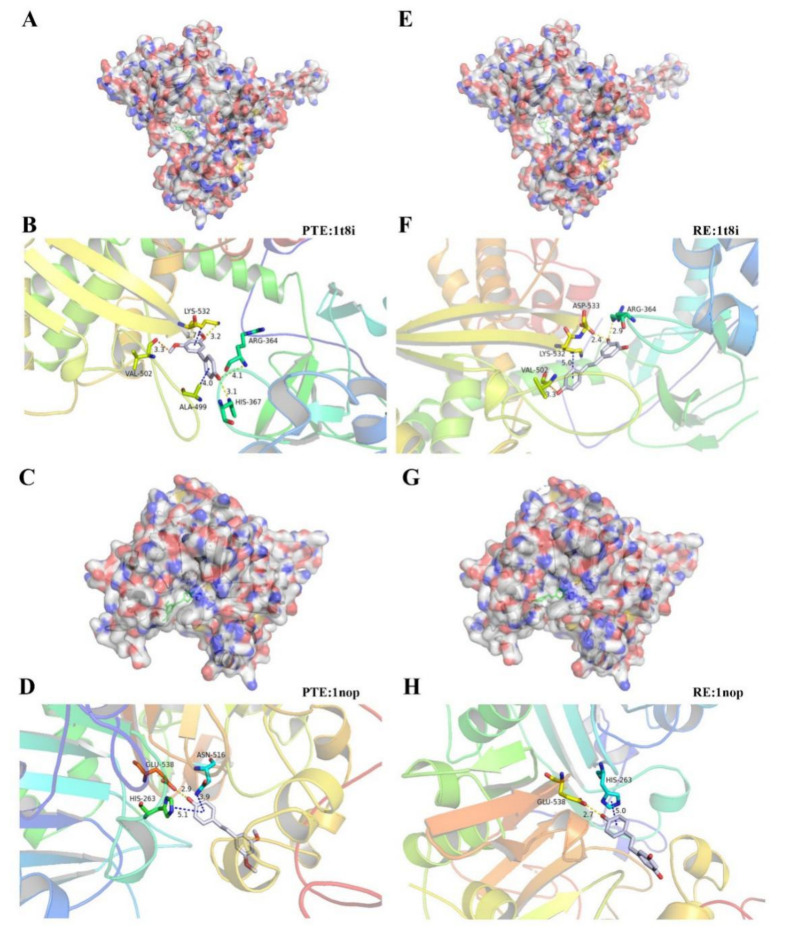
Molecular docking of PTE (**A**–**D**) and RE (**E**–**H**) in the active site of Top1 (PDB ID: 1T8I) and Tdp1 (PDB ID:1NOP). PTE and RE could enter the active center pocket of Top1 and Tdp1 and interact with the protein. Hydrogen bonds are indicated with yellow dashed lines, carbon-hydrogen bonds are indicated with orange dashed lines, and pi bonds are indicated with blue dashed lines. The carbons of compounds are colored in gray, nitrogen atoms in blue, and oxygen atoms in red.

In order to reveal the binding modes of PTE and RE with Top1 (PDB:1T8I) and Tdp1 (PDB:1NOP), respectively, molecular docking was investigated. PTE was found to enter the active center of Top1 and bound to active amino acids (Figure 5A) with a binding energy of −4.77 kcal/mol. As shown in Figure 5B, PTE interacted with Arg364, His367, Val502, Ala499, Lys532, and other residues of Top1. First, benzene Ring A and Ring B of main chain stilbene of PTE had interacted respectively with the residues of Ala499 and Lys532 to produceπ-Alkyl. The 3-OCH_3_ group and Lys532 formed hydrogen bonds, and the 4′-OCH_3_ group formed two hydrogen bonds with Arg364 and His367, respectively. In addition, the 5-OCH_3_ group and Val502 formed a carbon-hydrogen bond. RE entered the active center of Top1 to bind to active amino acids (Figure 5E) with a binding energy of −4.94 kcal/mol. As shown in Figure 5F, RE mainly interacted with Arg364, Val502, Lys532, and Asp533 of Top1 residues. The 5-OH formed two hydrogen bonds respectively with Arg364 and Asp533, and 4′-OH formed carbon-hydrogen bonds with Val502. In addition, there was a π-Alkyl (π-Alkyl) interaction between ring B of stilbene main chain of RE and the residue of Lys532. Lys532 is one of the important active amino acids of Top1, and it plays an important role in the process of Top1 binding to DNA and Top1 relaxing supercoiled DNA. The combination of RE or PTE with the active amino acid contributes to preventing the combination of DNA and Top1 enzyme, thus affecting the activity of Top1 enzyme.

Similarly, the molecular docking results of PTE and RE with Tdp1 showed that PTE entered the active center of Tdp1 and bound to active amino acids with a binding energy of −3.91 kcal/mol (Figure 5C). As shown in Figure 5D, PTE mainly interacted with His263, Asn516, Glu538, and other residues of Tdp1. The 4′-OCH_3_ and Glu538 formed a hydrogen bond. Theπ-πT-shaped interactions and π-donor hydrogen interactions were observed between styrene Ring B and His263 and Asn516 residues. RE entered the active center of Tdp1 and bound to active amino acids (Figure 5G) with a binding energy of −3.84 kcal/mol. As shown in Figure 5H, RE mainly interacted with His263 and Glu538 of Tdp1. The 4′-OH group of RE and Glu538 formed carbon-hydrogen bonds. A π-π T-shaped interaction was observed between Ring B of styrene and the residue of His263. His263 was one of the active amino acids of Tdp1 and it played an important role in capturing Top1-cc complexes, hydrolyzing 3′-phosphotyrosyl bonds, and releasing SSD. The combination of RE or PTE with active amino acids can effectively inhibit the function of Tdp1.

### 3.3. Effects of RE and PTE on Intracellular Top1 and Tdp1

#### 3.3.1. Effect of PTE and RE on Survival Rate of *Tdp*1-overexpressing and *Top*1- or *Tdp*1- Silenced CL187 Cells

We constructed *Tdp*1-overexpressing CL187 cell line (named *Top*1-over CL187), in which *Tdp*1 gene expression was 1481 times as high as that in the original cell line, and the cell survival rate of the constructed *Tdp*1-over line was significantly improved, which was almost twice as high as that of the original line, and the cells’ sensitivity to CPT was significantly reduced, but the cells exhibited a good response to FM. We also constructed *Top*1 gene-silencing CL187 cell line (named siRNA-*Top*1 CL187) in which *Top*1 gene expression was 19% of the original cell line, and the cell survival rate of constructed cell line was decreased, which was less than 50% of the original cell line. CPT lost its target in this cell, and hardly exhibited inhibitory effect on cell proliferation. FM inhibited the activity of Tdp1, thus blocking the DNA damage repair pathway of the cell and significantly enhancing the inhibitory effect on the cells. *Tdp*1-silencing cell line was constructed (named siRNA-*Tdp*1 CL187), in which *Tdp*1 gene expression was 28% of the original cell line. The cell survival rate of constructed siRNA-*Tdp*1 CL187 was reduced to about 80% of the original cell line. The sensitivity of cells to CPT was significantly increased, and the cell inhibition rate was about 90%, which might be due to the absence of Tdp1 enzyme repairing Top1 damage caused by CPT in the cell. FM lost its target, and thus failed to exert its efficacy (Figure S4, Table S3).

**Figure 6:**
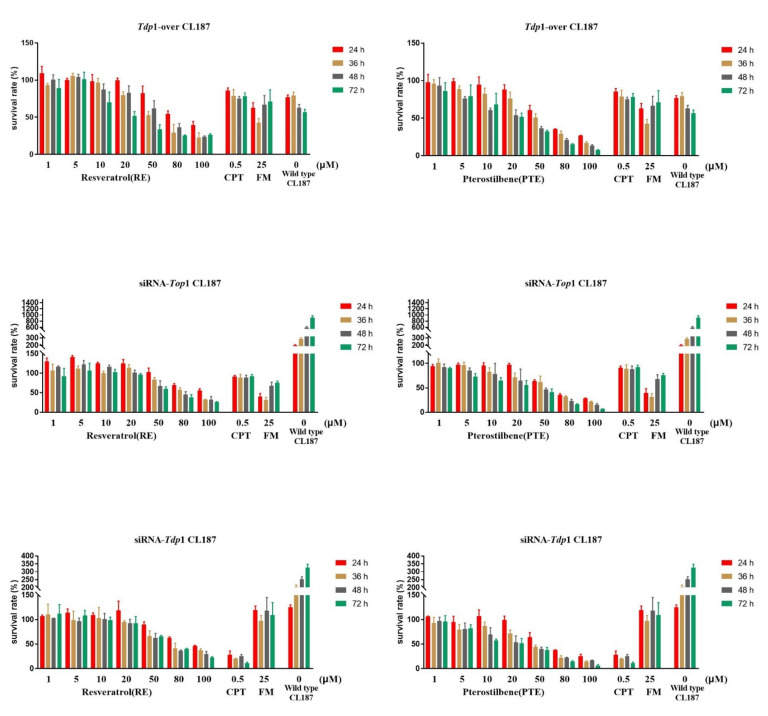
Cytotoxic results of *Tdp*1-overexpressing and *Top*1- or *Tdp*1- silenced CL187 cells incubated with PTE and RE for 24, 36, 48 and 72 h. Data are mean ± SD, *n* = 3.

PTE and RE at a concentration of 1–100 μM were incubated with the above-mentioned cells for 24, 36, 48, and 72 h, respectively, and the cell survival rate was shown in Figure 6. PTE and RE had good inhibitory effects on all the three gene-modulated cells with the calculated IC50 values shown in Table S2. The IC50 values of PTE acting on the three cell lines for 72 h were all around 20 μM. The IC50 value of *Tdp*1-over CL187 cells treated with RE for 72 h was 27.78 μM, and the IC50 values of siRNA-*Top*1 CL187 and siRNA-*Tdp*1 CL187 cells treated with RE for 72 h were both around 70 μM. The results showed that PTE was superior to RE in inhibiting these three types of cells.

#### 3.3.2. Pro-apoptotic Effects of PTE and RE on *Tdp*1-Overexpressing and *Top*1- or *Tdp*1- Silenced CL187 Cells

The pro-apoptotic effects of PTE and RE on the three constructed cells were investigated with the concentration of RE set as 20 μM and 50 μM and the concentration of PTE was set as 10 μM, 20 μM, and 50 μM. As shown in Figure S5, after the cells were incubated with RE and PTE for 48 h, the cell apoptotic proportion of *Tdp*1-over CL187 cells was 25.40% and 26.74% in RE-20 μM and 50 μM treatment group, and it was 26.80%, 31.81%, and 32.87% in PTE 10 μM, 20 μM, and 50 μM treatment groups. The cell apoptotic proportions of siRNA-*Top*1 CL187 cells were 29.36% and 34.80% in RE-20 μM and 50 μM treatment groups, and they were 25.29%, 29.42%, and 33.93% in PTE 10 μM, 20 μM, and 50 μM treatment groups. For siRNA-*Tdp*1 CL187 cells, it was 15.35% and 23.02% in RE-20 μM and 50 μM for the treatment groups, and 18.16%, 20.92%, and 24.38% in PTE 10 μM, 20 μM, and 50 μM treatment groups.

In addition, the nuclear shrinkage of the *Tdp*1-overexpressing and *Top*1- or *Tdp*1- silenced CL187 cells was investigated. As shown in Figure S6, in the blank control group, the nuclei were blue round or spindle-shaped with the identical size and large number. In the drug treatment groups, the number of cells was significantly reduced, and many nuclei were wrinkled and heavily stained, indicating that these cells had undergone apoptosis. With the increase in the drug concentrations, the number of cells was decreased, but the number of nuclei wrinkled and stained was increased. In the case of the same drug concentration, the number of wrinkled and heavily stained nuclei in the PTE treatment group was larger, while more nucleus fragments were observed in RE treatment group. These results indicated that both PTE and RE could induce apoptosis of various cells, and PTE had a significantly better ability to promote apoptosis than RE.

#### 3.3.3. Effects of PTE and RE on the Content of Intracellular Top1 and Tdp1 Protein and mRNA Expression

**Figure 7:**
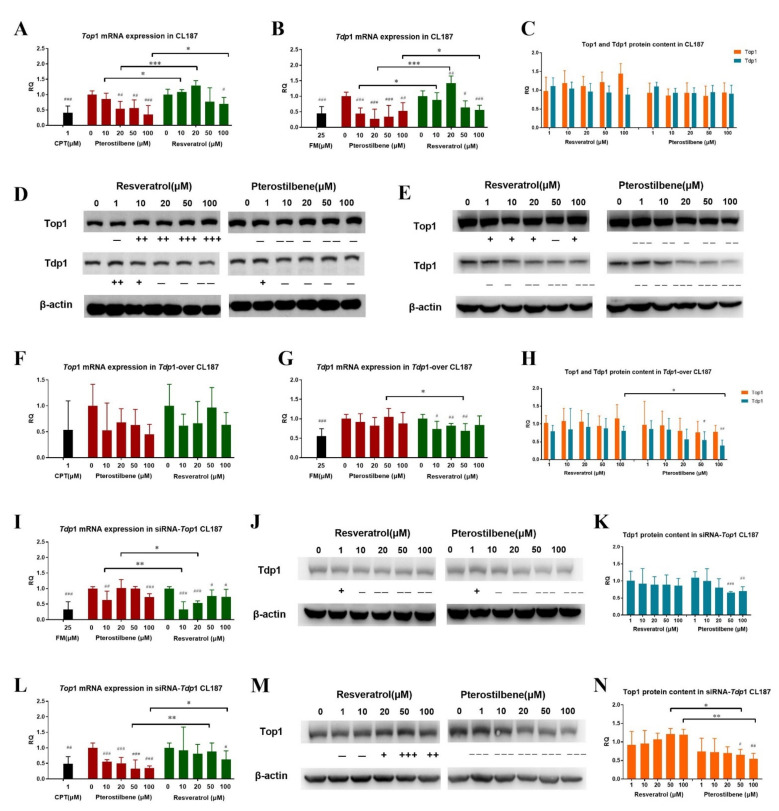
Effects of PTE and RE on intracellular Top1 and Tdp1 protein contents and mRNA expression after drug incubation for 6 h. The mRNA expression levels of *Top*1 and *Tdp*1 in CL187 cells (**A**,**B,C**). WB probing (**D**) and relative gray value analysis of WB results I for the protein content of Top1 and Tdp1 in CL187 cells. The mRNA expression levels of *Top*1 and *Tdp*1 in *Tdp*1-over CL187 cells (**F**,**G**). WB probiI(**E**) and relative gray value analysis of WB results (**H**) for the protein content of Top1 and Tdp1 in *Tdp*1-over CL187 cells. *Tdp*1 mRNA expression (**I**), WB probing (**J**) and relative gray value analysis of WB results (**K**) for the protein content of Tdp1 in siRNA-*Top*1 CL187 cells. *Top*1 mRNA expression (**L**), WB probing (**M**) and relative gray value analysis of WB results (**N**) for the protein content of Top1 in siRNA-*Tdp*1 CL187 cells. Loading was normalized based on the level of the internal control, β-actin. Compared with the control group, “+” or “−” was denoted when the change of gray value was between 0 and 10%, “++” or “− −” was denoted when the change of gray value was between 10–20%, “+++” or “− − −” was denoted when the change of gray value exceeded 20%. * *p* < 0.05, ** *p* < 0.01, and *** *p* < 0.001 vs. the RE treatment group. # *p* < 0.05, ## *p* < 0.01 and ### *p* < 0.001 vs. control group. ns, not significant. Data are mean ± SD, *n* = 3.

In order to further reveal anti-tumor mechanism of PTE and RE, the effect of PTE and RE on the Top1/Tdp1-mediated DNA repair pathway was further explored. The qPCR was performed to detect the transcription and expression of *Top*1 and *Tdp*1 in the cells, and WB assay was performed to detect the content of Top1 and Tdp1 in the cells. In wild-type CL187 cells, the expression levels of *Top*1 and *Tdp*1 were significantly inhibited in PTE-treated cells in a dose-dependent manner. In the RE treatment group, the intracellular *Top*1 and *Tdp*1 mRNA expression exhibited significant change only in the high-dose group (Figure 7A,B). We further detected the protein contents of Top1 and Tdp1 in the cells. As shown in Figure 7C,D, the protein content of Top1 did not decrease in the RE treatment group, whereas the protein content of Tdp1 was declined in a dose-dependent manner. In contrast, the protein contents of Top1 and Tdp1 were decreased in PTE treatment group, but neither in dese-dependent and significant manner.

In *Tdp*1-over CL187 cells, the intracellular mRNA expressions of *Top*1 and *Tdp*1 were decreased in RE-treatment group. In the PTE treatment group, the mRNA expression of *Top*1 was suppressed, but there was no significant difference, whereas the mRNA expression of *Tdp*1 did not change significantly (Figure 7F,G). The analysis of intracellular Top1 and Tdp1 protein content indicated that there was no significant change in the intracellular Top1 protein content in the RE treatment group, but the Tdp1 protein content was reduced. In the PTE treatment group, the contents of Top1 and Tdp1 proteins in the cells were reduced in dese-dependent manner (Figure 7E,H).

In PTE- and RE- treated siRNA-*Top*1 CL187 cells, the mRNA expression of *Tdp*1 was inhibited without obvious trend (Figure 7I). The protein content of Tdp1 was reduced in a dose-dependent manner (Figure 7J,K). In siRNA-*Tdp*1 CL187 cells, *Top*1 mRNA expression was significantly down-regulated with the increase of PTE concentration. The mRNA expression of *Top*1 was decreased but no significantly (except for 100 μM) in the RE treatment group (Figure 7L). However, the protein content of Top1 was significantly decreased only in PTE treatment group (Figure 7M,N).

### 3.4. Anti-Tumor Activity of PTE In Vivo

The above results indicated that the anti-tumor activity of PTE was better than that of RE in vitro. Therefore, we established two nude mice models of CL187 xenograft BALB/c nude mice models to evaluate the anti-tumor activity of PTE in vivo. 

#### 3.4.1. Antitumor Effect of PTE on Subcutaneous CL187 Xenograft Nude Mice

**Figure 8:**
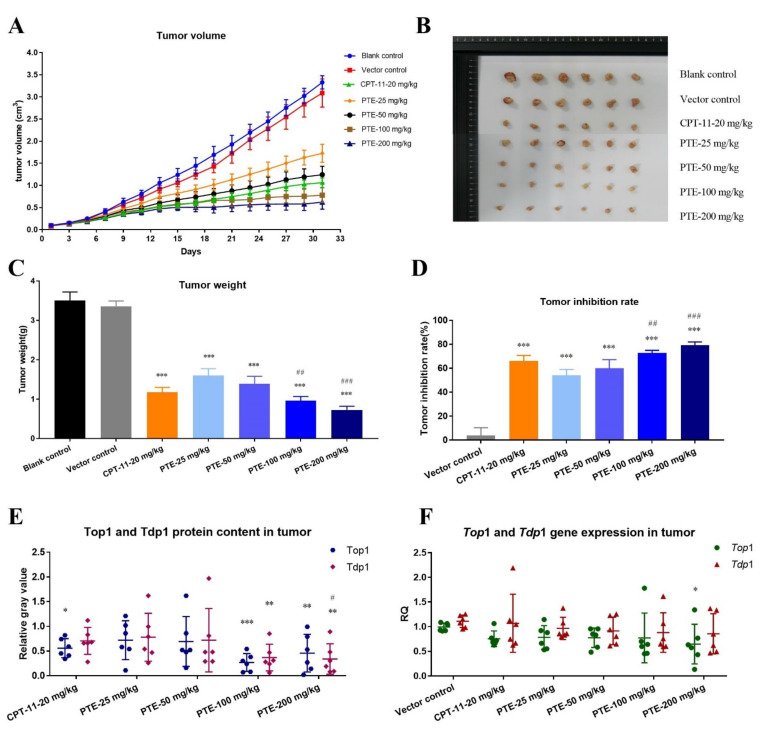
PTE suppressed tumor growth in subcutaneous CL187 xenograft nude mice. (**A**) Tumor volume. (**B**) Photograph of dissected tumor tissue. (**C**) Tumor weight. (**D**) Tumor inhibition Ie. (**E**) Relative gray value analysis of WB results for protein content of Top1 and Tdp1 in tumors tissues. Loading was normalized based on the level of the internal control, β-actin. (**F**) The expression levels of *Top*1 and *Tdp*1 in tumor tissues. Tumor of each animal were tested three times in parallel, with three replicates for each time. After the completion of the test, the average value was taken to draw the mRNA expression maps, and each point represented an animal. * *p* < 0.05, ** *p* < 0.01, and *** *p* < 0.001 vs. vector control. # *p* < 0.05, ## *p* < 0.01 and ### *p* < 0.001 vs. CPT-11 group. ns, not significant. Data are mean ± SD, *n* = 6.

During drug treatment, the animal’s body weight and tumor volume changes were recorded every two days. The results indicated that the animal’s body weight steadily increased (Figure S7A), and the tumor’s development was controlled by a drug (Figure 8A). The tumor volume in drug treatment group was significantly smaller than that of the control group. After the animals were treated for 31 days, we dissected the tumor tissues, took pictures, weighed, and calculated the tumor growth inhibition rate (TGI) (Figure 8B–D, Table 2). The TGI of the vector control group was 3.77 ± 6.57%, indicating that the solvent had no effect on tumor treatment. In the positive control group, the TGI of the CPT-11 group was 66.24 ± 4.50%. In the PTE treatment group, the TGI of the 35, 50, 100 and 200 mg/kg dose groups were 54.24 ± 4.80%, 59.92 ± 7.32%, 72.70 ± 2.23%, 79.14 ± 2.85%, respectively. These results indicated that PTE had a strong anti-tumor efficacy in vivo. During the entire treatment process, the animals in the CPT-11 treatment group had severe diarrhea, lassitude, and other adverse reactions, while the nude mice in the PTE treatment group did not have the above-mentioned adverse reactions. These results indicated that PTE not only had a good anti-tumor effect in vivo, but also had the advantage of low toxicity.

There was no significant difference between male and female animals during treatment. The anti-tumor mechanism of PTE was further explored by detecting the effect of PTE on the DNA repair pathway mediated by Top1/tdp1 in vivo. WB assay results indicated that the protein contents of Top1 and Tdp1 in animal tumor tissues were decreased significantly (Figure 8E and Figure S7), especially in the positive control group and the high-concentration PTE treatment group. At the same time, the mRNA expression of *Top*1 and *Tdp*1 in tumor tissues was inhibited (Figure 8F).

#### 3.4.2. Antitumor Effect of PTE on Orthotopic CL187 Xenograft Nude Mice

In order to investigate whether PTE’s different administration methods affected its anti-tumor effect in vivo, intraperitoneal injection and intragastric administration were performed. The dose of PTE intraperitoneal injection was 100 mg/kg, and the dose of PTE intragastric administration was 500 mg/kg. The dose setting was based on the results of our previous pharmacokinetics study on PTE. The results indicated that the bioavailability of PTE to animals is the same under these two drug administrations.

**Figure 9:**
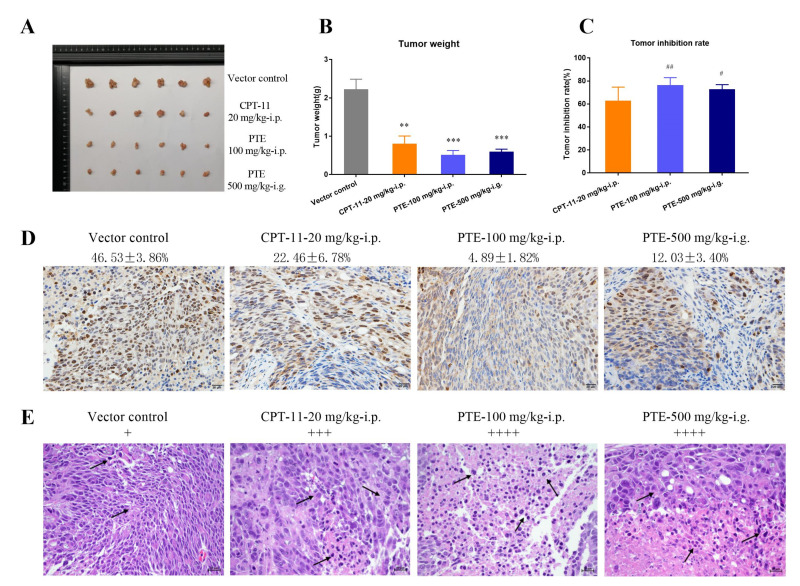
Inhibitory effect of PTE on tumor growth in orthotopic CL187 xenograft nude mice. (**A**) Photograph of dissected tumor tissue. (**B**) Tumor weight. (**C**) Tumor inhibition rate. ** *p* < 0.01, and *** *p* < 0.001 vs. vector control. # *p* < 0.05, and ## *p* < 0.01 vs. CPT-11 group. ns, not significant. Data are mean ± SD, *n* = 6. (**D**) Immunohistochemical analysis of Ki67 staining for tumors; the scale bar represents 20 μm. The yellow cells are those labeled IKi67. (**E**) HE assays for tumors; the scale bar represents 20 μm. The degree of tumor necrosis was expressed as +, and + represents the ratio of tumor necrosis area to total tumor tissue <1/4; ++ indicates the ratio was between 1/4 and 1/2; +++ denotes the ratio between 1/2 and 3/4, and ++++ indicate the ratio >3/4. The areas indicated by the black arrow are apoptotic cells and inflammatory cells in the tumor necrotic area.

After the animals were treated for 21 days, we dissected the tumor tissues, took pictures, weighed, and calculated the TGI value (Figure 9A–C, Table 3). The TGI of the CPT-11 treatment group was 62.91 ± 11.78%. The TGI of the 100 mg/kg PTE intraperitoneal injection group was 76.57 ± 6.34%, and the TGI of the 500 mg/kg PTE intragastric administration group was 72.79 ± 4.06%. The Ki67 immunohistochemical analysis of tumor tissues (Figure 9D) showed that PTE significantly inhibited tumor proliferation. The Ki67 labeling rates in each group were 4.89 ± 1.82% (i.p.) and 12.03 ± 3.40% (i.g.), which were significantly lower than those in CPT -11 treatment group (22.46 ± 6.78%). The tumor tissue was stained with HE (Figure 9E), and staining results indicated that in the PTE treatment group, the tumor tissues of the animals exhibited a large area of necrosis (+ + + +). The above results showed that both PTE intraperitoneal injection and PTE intragastric administration had the same anti-tumor effect in animals under the same bioavailability.

**Figure 10:**
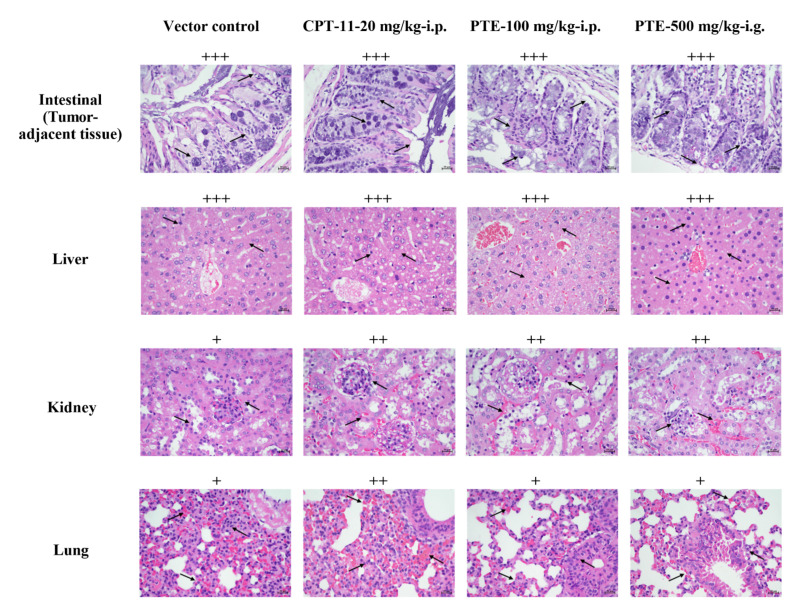
HE assays of paracancerous colon tissues, liver, kidney and lung. The scale bar represents 20 μm. The degree of organ injury is expressed by +. In intestinal tissue, four indexes are mainly evaluated including gland atrophied, epithelial thinning, goblet cell loss or abscission, and gland loss. Each occurrence of one of them results in an additional +. In the liver, + indicates <5% hepatocyte adipose change, no inflammatory cell infiltration, hepatocyte necrosis, and hepatocyte balloon-like change. ++ means hepatocyte fat change between 5% and 33%, scattered inflammatory cell infiltration, local hepatocyte necrosis, and rare hepatocyte balloon-like change. +++ indicates that hepatocyte fat change between 34% and 66%, focal inflammatory cell infiltration, local or diffuse hepatocyte necrosis, and hepatocyte balloon-like change was less than 5. ++++ indicates >66% fatty changes in liver cells, severe inflammatory cell infiltration, necrosis area of liver cells >1/2 of hepatic lobular area, and >5 hepatocyte balloon-like changes in liver cells. In the kidney, four indexes are mainly evaluated including glomerular size change, glomerular cell degeneration or necrosis, kidney tubules swelling and congestion, and inflammatory cell infiltration. Each occurrence leads to an additional +. In the lung four indexes are evaluated: alveolar wall thickening, alveolar congestion, alveolar structural destruction (bullae), and inflammatory cell infiltration. Each occurrence of one of them results in an additional +. Typical lesions in each organ are indicated by black arrows.

The liver, kidney, lung, and paracancerous colon tissues of nude mice were analyzed by HE staining (Figure 10). Observation of the nude mice’s colon sections revealed the nude mice’s colon tissue glands were intact, but they were arranged loosely with various degrees of gland atrophy. Their intestinal structure remained intact, but the intestinal epithelium was thinned, and cancer cells and tumor lesions were observed in the intestine. The drug treatment group exhibited no obvious advantage over the solvent control group, which may be related to the sampling location too close to the tumor. Since the tumor has not been completely eliminated, tumor lesions were still observed in the intestinal tissues adjacent to the cancer. The observation of liver slices of nude mice indicated that the livers of nude mice were damaged to different degrees, that the hepatic lobular structure of the liver tissue was normal without necrosis. The inflammatory cells were observed to be distributed around the hepatic lobular vessels and/or in the liver parenchyma.

In addition, tumor metastases were observed in the liver parenchyma of the solvent control group. However, the volume of these tumor metastases was small, and they did not invade the liver parenchyma. The capsule was intact. In the PTE group, a small amount of scattered cancer cells were observed in the liver parenchyma, but they did not form metastases lesions. These observations indicated that the PTE treatment caused no additional damage to the animal’s liver, and it inhibited the development and metastasis of tumor cells. Observation of the kidney sections of nude mice indicated that the kidneys of nude mice exhibited various degrees of inflammatory cell infiltration, that kidney tubules swelled in individual animals, and that renal interstitium had no obvious hyperemia. In the high-power lens, inflammatory cell infiltration was observed. In the PTE treatment group, the inflammatory response of animals was more obvious, but there was no difference in the inflammatory response of animals between the PTE and CPT-11 groups. These results suggested that the drug treatment might have effect on the kidney and even cause kidney inflammation. Observation of the lung slices of nude mice demonstrated that the alveolar wall and bronchus of nude mice were structurally intact, that there were no secretions in the lumen, and that alveolar wall was thickened to different degrees. These phenomena were more obvious in the animals in the CPT-11 treatment group, and they were relatively less serious in PTE treatment group and in solvent control group. These above observations suggested that lung inflammation was common in tumor-bearing nude mice, that PTE had no significant effect on lung inflammation, while CPT- 11 could aggravate the inflammatory response.

## 4. Discussion

CPT and its derivatives target mammalian Top1 enzymes and are currently one of the most effective new anti-cancer drugs with good therapeutic effects on a variety of malignant tumors [40,41]. However, there are many defects in the clinical use of CPT derivatives, which seriously limit their application [42]. We found that PTE and RE can down-regulate the protein content and RNA expression of Top1 and Tdp1, which can be developed as a new generation of Top1 inhibitors. 

Both PTE and RE are derivatives of stilbene, and they have similar characteristics in anti-tumor efficacy, but there are great difference in their drug efficacy [24]. This study found that PTE had better cell permeability than RE, and it could quickly enter the nucleus and mitochondria with abundant Top1 enzymes and Tdp1 enzymes in the cells, and PTE had a higher concentration in these two organelles, enabling a variety of functional enzymes to be exposed to the high concentration of PTE, which was conducive to the corresponding function of PTE. This finding provides evidence for the hypothesis that PTE and RE could directly act on the target protein. 

Compared with RE, PTE has been reported to have better photostability and thermostability, higher bioavailability, slower metabolic rate, and longer half-life period in various in-vivo tests, therefore, PTE has also shown advantages over RE [24,27,43,44,45]. In this study, the analysis of the structural characteristics of these two compounds revealed that stilbene was the main chain for both PTE and RE, that RE had three hydroxyls at the 3, 4′, and 5 positions, and it was easily hydrolyzed in vivo, which might explain its poor stability. PTE had a hydroxyl group at the 4′ position and two methoxy groups at the 3 and 5 positions. The presence of the methoxy group made the compound stable to avoid the oxidative hydrolysis reactions, so that the compound could maintain its exposure amount and exposure time in the cells or in vivo, which was conducive to the exertion of the drug efficacy [46]. Considering this feature, we can conduct further corresponding modifications or transformations on the basis of stilbene to obtain more efficient and low-toxic new structure compounds. This is also the ongoing work of our research group.

Since it is easy to produce drug resistance against CPT and its derivatives in clinical applications, many scholars have explored the mechanism of drug resistance and established several hypotheses. The first hypothesis lies in the change of Top1 such as the decrease in *Top*1 expression or its mutation [47]. We also confirmed this hypothesis in *Top*1-silenced cell line. When *Top*1 gene was silenced, the sensitivity of cells to CPT was decreased. Another hypothesis maintains that reducing or removing the cleavable complex formed by the interaction between CPT and Top1 leads to the generation of cell drug resistance [48], for example, overexpression of *Tdp*1 can protect cells from damage by Top1 inhibitors, while Tdp1 inactivation or the down-regulation of its expression can increase the sensitivity of the cells to the Top1 inhibitor [49]. Similarly, our results indicated that in *Tdp*1-overexpressing cell line we designed, the sensitivity of the cells to CPT was significantly reduced, while it was significantly increased in the *Tdp*1-silenced cell line. We found that PTE and RE can inhibit the proliferation of *Tdp*1-overexpressing and *Top*1-or *Tdp*1-silenced CL187 cells, reduced the transcription and expression levels of Top1 and Tdp1 in these cells. In conclusion, PTE and RE can affect the DNA damage repair pathway mediated by Top1/Tdp1 in cells, thus causing apoptosis or death of cells. 

In addition, the drug resistance to Top1 inhibitors in application might be related to the cross-drug resistance or multi-drug resistance (MDR) caused by the overexpression of drug efflux membrane transporters ABCG2 and ABCB1 (P-gp) [50,51,52]. Previous studies have shown that the reduction in CPT accumulation promotes the production of cell resistance in vivo [53]. P-gp is a transporter protein that is active on the surface of the gastrointestinal tract. Many anti-cancer drugs are substrates of P-gp. When taken orally, these drugs are transported back to the gastrointestinal tract by P-gp, which limited the absorption process of the drug [54]. P-gp overexpression is the classic mechanism of MDR production [55]. PTE and RE can down-regulate the expression of the P-gp-encoding gene *MDR*1, thus regulating the post-transcriptional modification process of *MDR*1, affecting the translation of P-gp and inhibiting the effect of P-gp, eventually reversing the resistance to multiple anti-tumor drugs [29,56,57]. It has also been reported that compounds with stilbene structure (stilbene) had a certain effect on the expression and function of P-gp [55], which provides a new perspective for chemotherapeutic drug in combination with styrene structure compounds to relieve drug resistance.

In addition, PTE can inhibit tumor cells through modulation of Akt, mitogen-activated protein, metastasis-associated protein 1, oxidative stress, endoplasmic reticulum stress, mitochondrially derived apoptosis, autophagy-dependent manner autophagy, cell cycle arrest and non-apoptotic pathways [28,30,31,32,58,59,60,61,62]. In conclusion, in addition to inhibiting the Top1/Tdp1 pathway by PTE and RE, there are other ways to inhibit tumor cells, which need to be further discovered.

There are some problems with existing Top1 inhibitors in clinical application such as serious side effects. For example, when topotecan is used, adverse reactions such as fatigue, alopecia, bone marrow cell destruction, and anemia may occur [63]. Irinotecan may cause fetal malformations; thus, contraception has to be taken during its clinical application [64]. In our research, we found that PTE caused less damage to animals, that PTE caused no side effects such as diarrhea, lethargy, neurotoxicity even in the high-dose group. Combined with previous research reports, it could be concluded that both PTE and RE, as plant-derived polyphenolic compounds, have high safety [65,66]. In addition, some previous studies have shown that PTE is not toxic to rodents, nor is it toxic to normal cells in vitro [67]. In summary, PTE can not only exert the better anti-tumor efficacy, but also avoid the production of toxic effects and side effects, thus PTE is promising to be developed to be an anti-tumor drug with high safety and better efficacy.

## 5. Conclusions

This study indicates that PTE and RE are inhibitors of Top1, which can effectively inhibit the DNA damage repair pathway mediated by Top1 and Tdp1, and induce apoptosis and death of a variety of colorectal cancer cells. PTE has a more stable structure and superior pharmacokinetic properties; thus, it has a better anti-tumor effect in vivo with a higher ability to inhibit Top1 and Tdp1. PTE is safe and has no serious side effects even in large doses. With the same bioavailability, whether intraperitoneally injected or intragastrically administrated, PTE has the same anti-tumor efficacy in vivo, and the oral administration method is safer and more convenient. Therefore, PTE has a greater potential to be further developed as an anti-tumor drug for the treatment of colorectal cancer through Top1/Tdp1-mediated DNA repair pathway.

## 6. Patents

Some results of this research have been applied for patent in China. The patent number is 202010595033.

## Figures and Tables

**Table 1:** The IC50 values of PTE and RE in colorectal cells. Data are mean ± SD, *n* = 3.

Compound	Time (h)	IC50 (μM)					
		HCT-8	SW480	LoVo	COLO205	CL187	HCT-116
Pterostilbene	24	29.91 ± 10.64	112.6 ± 23.97	106.1 ± 28.61	44.35 ± 16.70	24.21 ± 11.64	31.17 ± 10.21
	48	25.43 ± 8.25	35.73 ± 9.32	14.15 ± 4.84	24.5 ± 9.13	13.82 ± 4.70	15.44 ± 4.49
	72	25.02 ± 6.62	40.50 ± 10.76	11.95 ± 4.01	13.56 ± 6.10	14.42 ± 2.93	21.43 ± 6.79
Resveratrol	24	36.26 ± 13.33	62.13 ± 15.63	165.5 ± 33.70	49.71 ± 22.54	25.97 ± 8.29	39.98 ± 14.48
	48	32.15 ± 9.15	41.83 ± 7.90	44.74 ± 10.27	29.73 ± 9.72	19.41 ± 8.47	18.87 ± 4.05
	72	26.22 ± 8.37	47.98 ± 12.36	16.82 ± 5.13	23.80 ± 10.02	23.23 ± 6.06	33.14 ± 6.03

**Table 2:** The tumor inhibition rate of each administration group in subcutaneous CL187 xenograft nude mice (Mean ± SD, *n* = 6).

Group	Tumor Volume (cm^3^)	Relative Tumor Proliferation Rate (T/C, %)	Tumor Weight (g)	Tumor Inhibitory Rate (TGI, %)
Blank control	3.328 ± 0.149	--	3.50 ± 0.22	--
Vector control	3.088 ± 0.318	100.34%	3.36 ± 0.13	3.77 ± 6.57%
CPT-11 20 mg/kg	1.062 ± 0.11 ***	34.46%	1.18 ± 0.12 ***	66.24 ± 4.50% ***
PTE 25 mg/kg	1.725 ± 0.203 **	53.53%	1.60 ± 0.17 ***	54.24 ± 4.80% ***
PTE 50 mg/kg	1.243 ± 0.189 ***	39.56%	1.39 ± 0.19 ***	59.92 ± 7.32% ***
PTE 100 mg/kg	0.779 ± 0.168 ***	24.20%	0.96 ± 0.11 ***	72.70 ± 2.23% ***
PTE 200 mg/kg	0.623 ± 0.158 ***	20.71%	0.73 ± 0.09 ***	79.14 ± 2.85% ***

** *p* < 0.01, and *** *p* < 0.001 vs. vector Control. ns, not significant. Data are mean ± SD, *n* = 6.

**Table 3:** The tumor inhibition rate of each administration group on in situ colorectal cancer tumor in nude mice (mean ± SD, *n* = 6).

Group	Tumor Weight(g)	Tumor Inhibitory Rate (%)
Vector control	2.23 ± 0.26	--
CPT-11 20 mg/kg-i.p.	0.81 ± 0.20 **	62.91 ± 11.78%
PTE 100 mg/kg-i.p.	0.51 ± 0.12 ***	76.57 ± 6.34% ##
PTE 500 mg/kg-i.g.	0.60 ± 0.06 ***	72.79 ± 4.06% #

** *p* < 0.01, and *** *p* < 0.001 vs. vector control. # *p* < 0.05, and ## *p* < 0.01 vs. CPt-11 group. ns, not significant. Data are mean ± SD, *n* = 6.

## Data Availability

Data are contained within the article or Supplementary Material.

## Supplementary Materials

The following are available online at https://www.mdpi.com/article/10.3390/cancers13164002/s1, Figure S1: *Tdp*1 overexpression plasmid information. Figure S2: Anti-tumor effects of PTE and RE on CL187, COLO 205, HCT-8, HCT-116, LoVo and SW480 inhibition rates against cancer cells during various periods. Figure S3: Survival rate to proliferative non-tumor cells for 24, 48 and 72 h treated with PTE or RE. Figure S4: Survival rate to genetically modified CL187 cells for 24, 48 and 72 h treated with medium, CPT and FM. Figure S5: *Tdp*1-over CL187 cells, siRNA-*Top*1 CL187 cells and siRNA-*Tdp*1 CL187 cells were treated with different concentrations of PTE and RE for 48 h, followed by flow cytometry. Figure S6: *Tdp*1-over CL187 cells, siRNA-*Top*1 CL187 cells and siRNA-*Tdp*1 CL187 cells were treated with different concentrations of PTE and RE for 48 h, and stained cells were observed under a multimode microplate detection and cell imaging system (×400). Figure S7: (A) Body weight of the subcutaneous CL187 xenograft nude mice. (B) Photograph of the mice in last day. (C) WB probing for Top1 and Tdp1 in tumor tissues. Figure S8: (A) Body weight of orthotopic CL187 xenograft nude mice. (B) Photograph of the mice in last day. Table S1: IC50 of PTE and RE in proliferative non-tumor cells. Table S2: IC50 of resveratrol or pterostilbene on *Tdp*1-over CL187 cells, siRNA-*Top*1 CL187 cells and siRNA-*Tdp*1 CL187 cells. Table S3. The mRNA expression on Tdp1-over CL187, siRNA-Top1 CL187 and siRNA-Tdp1 CL187 cell lines.

## Abbreviations

RE, resveratrol; PTE, pterostilbene;Top1, topoisomerase 1; Tdp1, tyrosyl-DNA phosphodiesterase 1; CCK-8, cell counting kit-8; DMSO, dimethyl sulfoxide; DM’M, Dulbecco’s mo’ified Eagle’s medium; RPMI 1640, Roswell Park Memorial Institute 1640; DMEM/F’2, Dulbecco’s Modified Eagle Medium/Nutrient Mixture F-12; FITC, fluorescein isothiocyanate; GAPDH, glyceraldehyde-3-phosphate dehydrogenase; CPT, camptothecin; CPT-11, irinotecan; SDS-PAGE, sodium dodecyl sulphate-polyacrylamide gel electrophoresis; FM, furamidine dihydrochloride; DEPC, diethylpyrocarbonate water; ECL, enhanced chemiluminescence; TAE, Tris-acetate-EDTA; HP-β-CD, 2-Hydroxypropyl-β-cyclodextrin; i.p., intraperitoneal injection; i.g., intragastric administration; qPCR, real-time fluorescence quantification; WB, western blot; DAB, 3,3’-diaminobenzidine; HE, hematoxylin and eosin; TGI, tumor growth inhibition rate.
