# Supplementary material for: Effect of Pterostilbene, a Natural Derivative of Resveratrol, in the Treatment of Colorectal Cancer through Top1/Tdp1-Mediated DNA Repair Pathway
Source: Cancers (Basel). 2021 Aug 9;13(16):4002. doi: 10.3390/cancers13164002 (PMC8391236; doi:10.3390/cancers13164002)
Supplement: Supplementary file 1 [file cancers-13-04002-s001.zip › cancers-1271510-supplementary.pdf]

Supporting Information for
Original article

Effect of pterostilbene, a natural derivative of resveratrol, in the Treatment of Colorectal Cancer through Top1/Tdp1-Mediated DNA Repair Pathway

Yutian Zhang, Ying Li, Changcheng Sun, Xiang Chen, Luyao Han, Tingqiang Wang, Jinfeng Liu, Xijing Chen*, and Di Zhao*

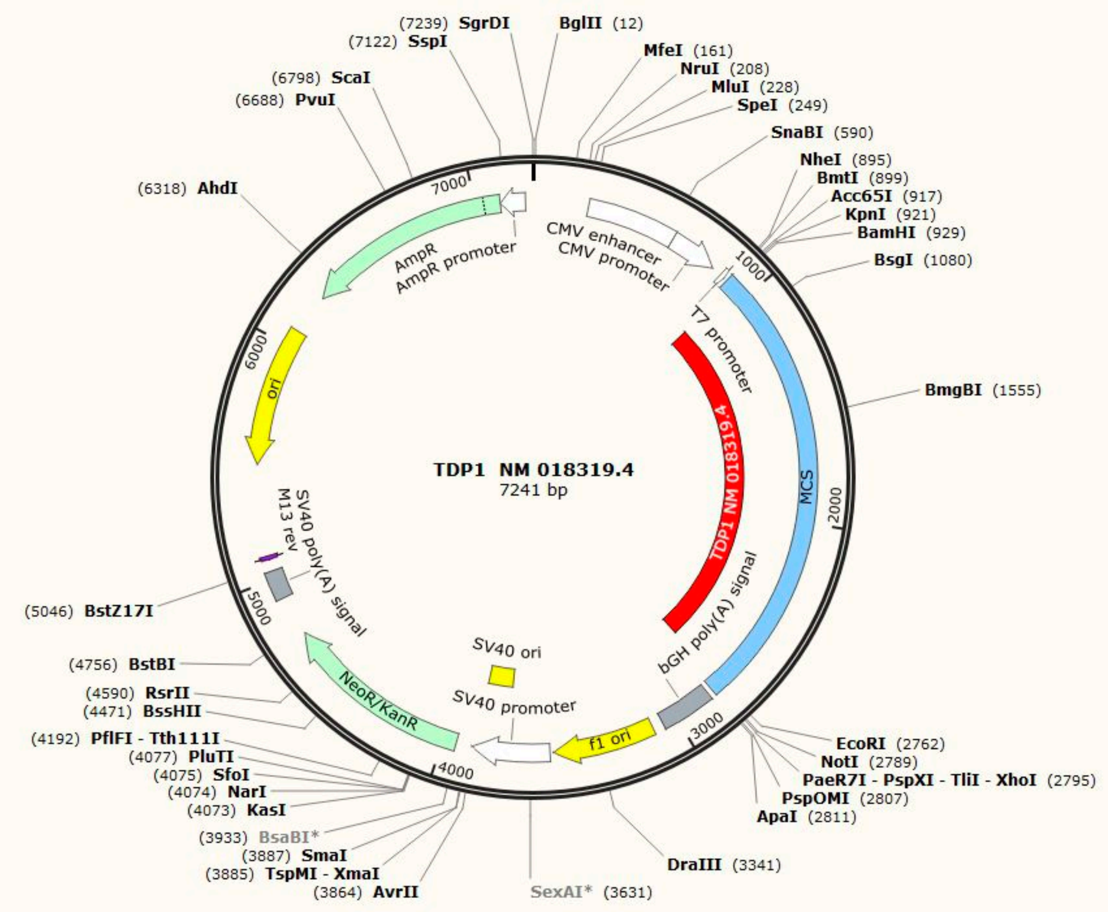

Figure S1. *Tdp*1 overexpression plasmid information. Gene name: TDP1 NM018319.4. Vector: pcDNA3.1(+). Clone site: *BamHI-EcoRI*.

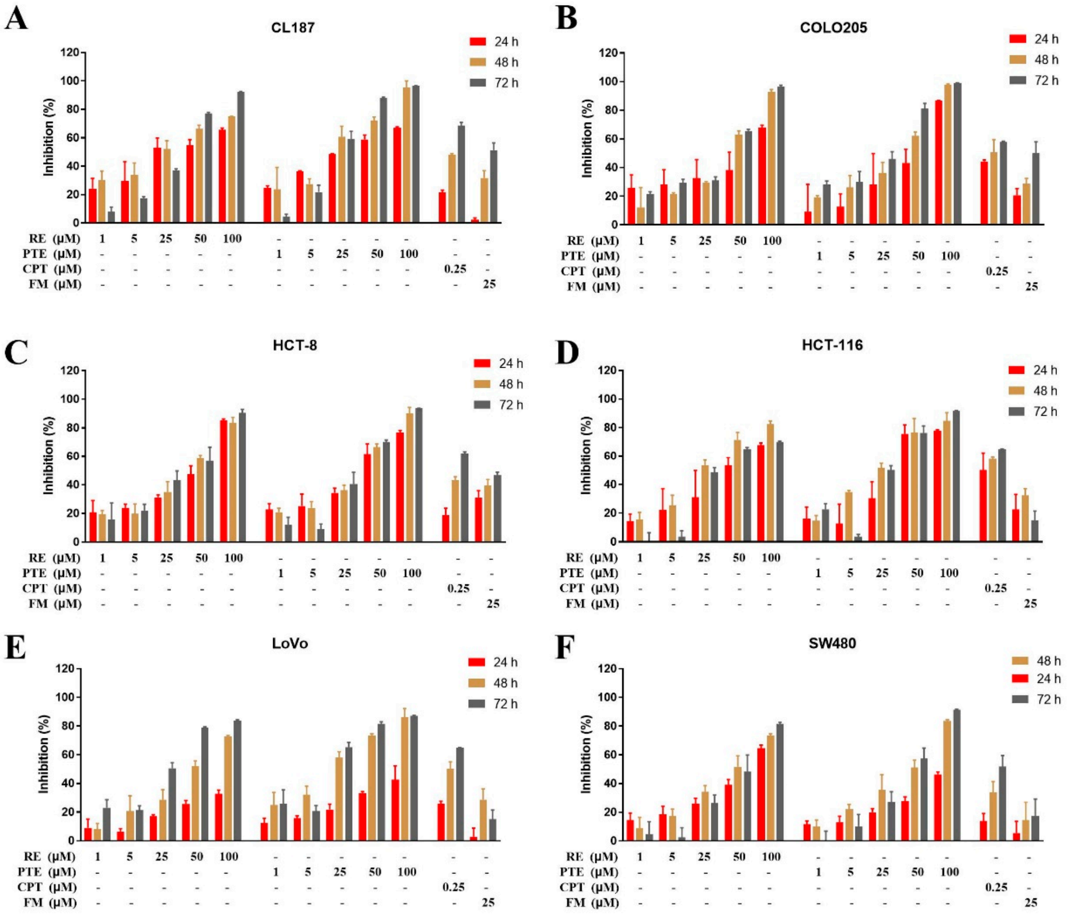

Figure S2. Anti-tumor effects of PTE and RE on CL187 (A), COLO 205(B), HCT-8 (C), HCT-116 (D), LoVo (E) and SW480 (F) inhibition rates against cancer cells during various periods. Data are mean ± SD, n=3.

Table S1. IC50 of PTE and RE in proliferative non-tumor cells. Data are mean ± SD, n = 3.

IC50	HCoEpiC-			HFL-1			L02		
Time(h)	24	48	72	24	48	72	24	48	72
PTE	443.8± 51.89	249.7± 68.21	83.43± 29.46	338.6± 30.56	315.1± 73.94	130.2± 28.95	478.4± 70.72	248.0± 39.42	148.1± 28.05
RE	739.2± 59.76	687.5± 113.2	137.9± 17.44	245.1± 62.90	224.6± 40.72	116.7± 40.31	534.5± 40.30	392.5± 69.20	311.2± 30.31

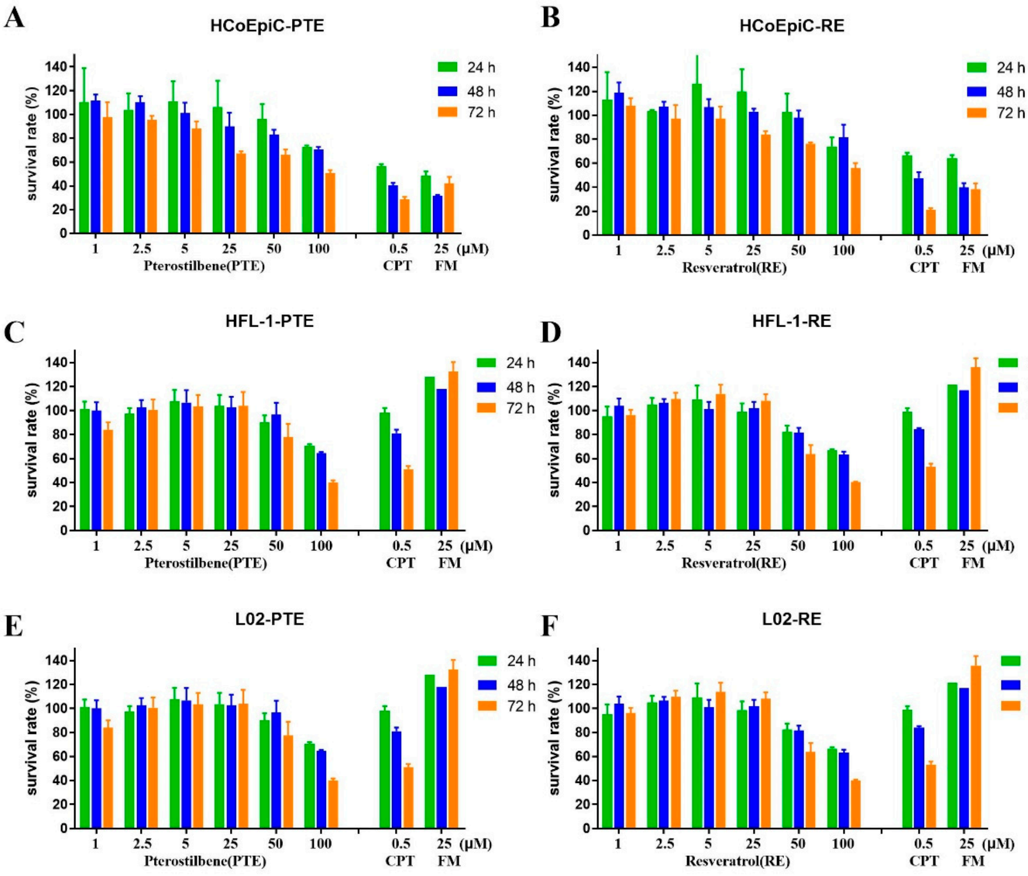

Figure S3. Survival rate to proliferative non-tumor cells for 24, 48 and 72 h treated with PTE or RE. Data are mean ± SD, n = 3.

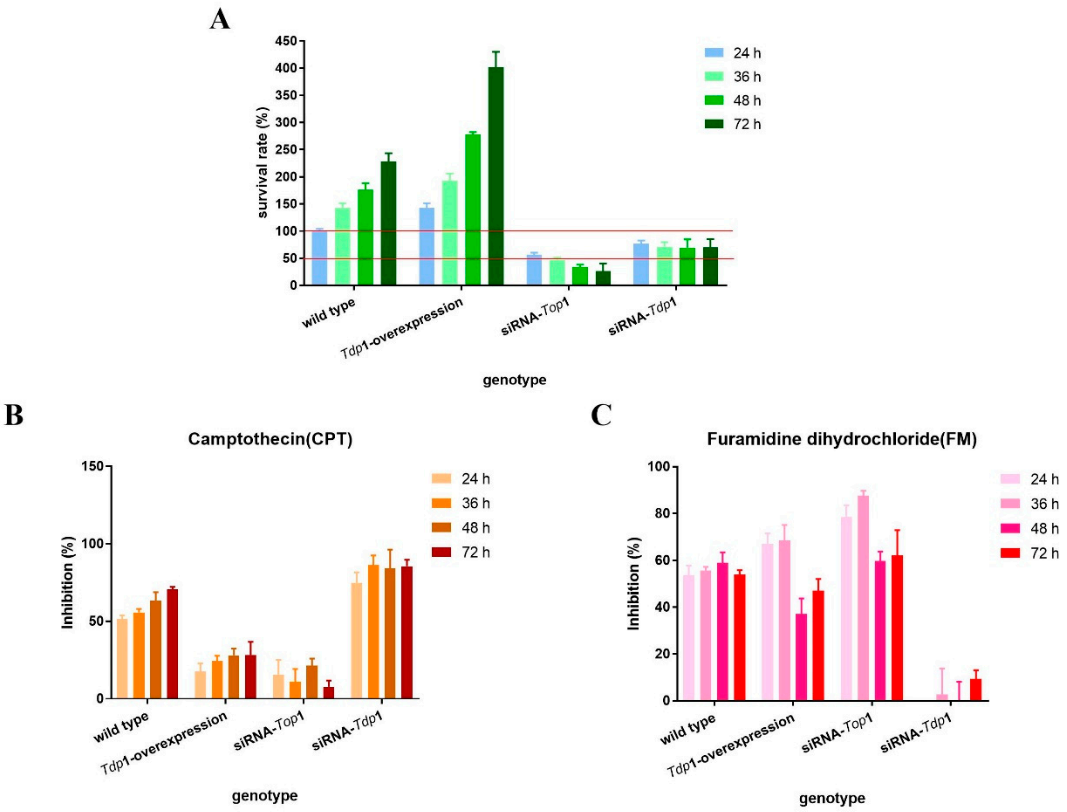

Figure S4. Survival rate to genetically modified CL187 cells for 24, 48 and 72 h treated with (A) medium, (B) camptothecin (CPT) and (C) furamidine dihydrochloride (FM). Data are mean ± SD, n = 3.

Table S2. IC50 of resveratrol or pterostilbene on *Tdp*1-over CL187 cells, siRNA-*Top*1 CL187 cells and siRNA-*Tdp*1 CL187 cells. Data are mean ± SD, n = 3.

IC50（μM）	Time(h)	Tdp1-over CL187	siRNA-Top1 CL187	siRNA-Tdp1 CL187
Resveratrol	**24**	118.90±30.97	312.30±83.70	182.20±70.80
	**36**	51.18±11.72	127.50±25.63	85.01±26.91
	**48**	59.04±12.87	96.82±36.19	70.33±16.71
	**72**	27.78±6.31	71.43±21.49	71.75±21.61
Pterostilbene	**24**	63.02±13.58	69.96±16.20	74.20±21.79
	**36**	41.12±7.22	49.04±10.09	34.19±7.33
	**48**	20.29±3.13	31.89±8.11	24.77±4.62
	**72**	19.14±4.08	20.16±4.10	18.38±3.78

Table S3. The mRNA expression on *Tdp*1-over CL187, siRNA-*Top*1 CL187 and siRNA-*Tdp*1 CL187 cell lines.

Cell lines	*Top*1 mRNA	*Tdp*1 mRNA
**Wild type CL187**	1.01 ± 0.15	1.00 ± 0.11
***Tdp*1-over CL187**	N/A	1481.8 ±265.24
**Vector plasmid CL187**	N/A	0.95 ± 0.06
**siRNA-NC CL187**	1.11 ± 0.15	1.08 ± 0.06
**siRNA-*Top*1 CL187**	0.19 ± 0.02	N/A
**siRNA-*Tdp*1 CL187**	N/A	0.28 ± 0.03

N/A：Not tested

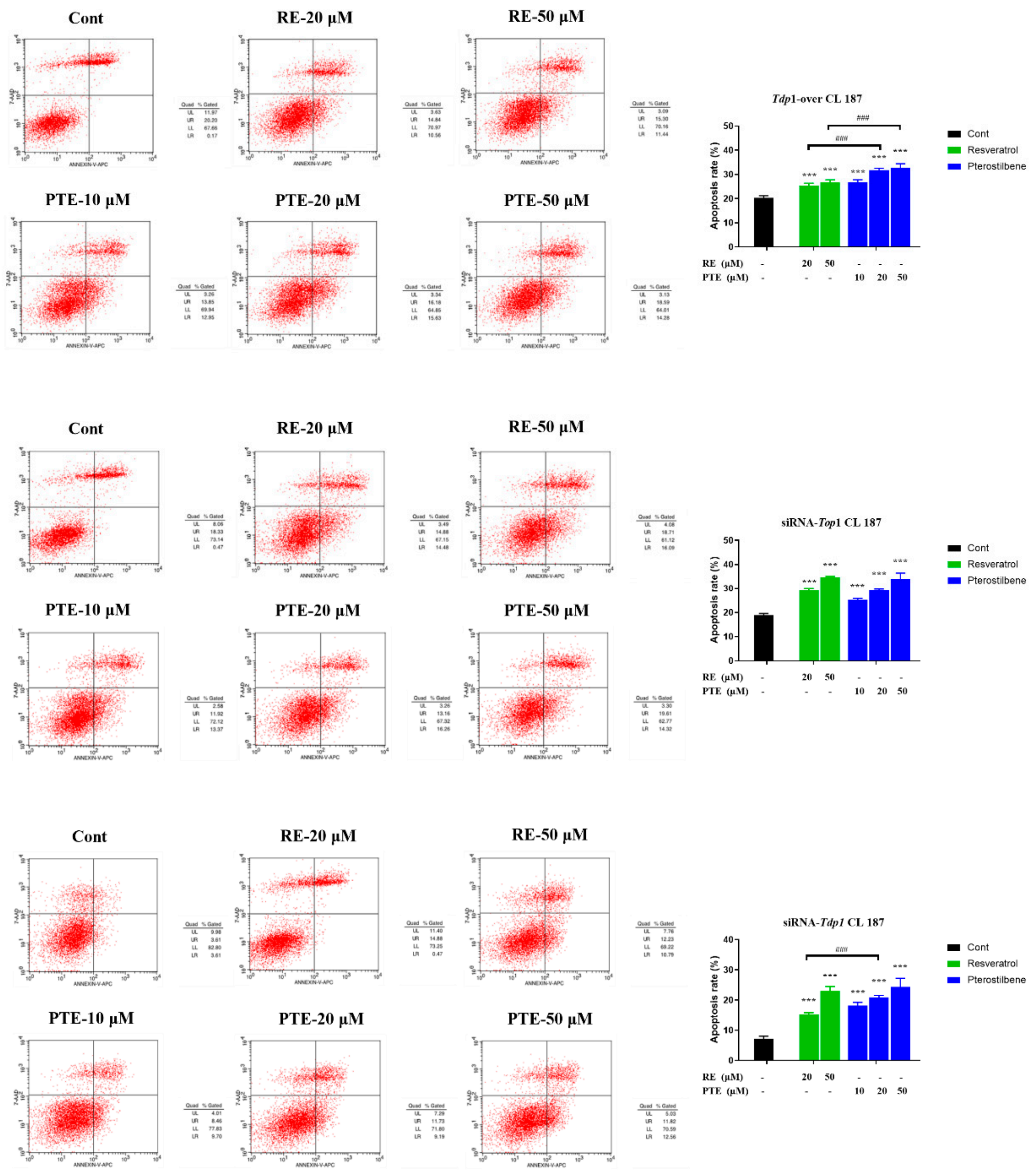

Figure S5. *Tdp*1-over CL187 cells, siRNA-*Top*1 CL187 cells and siRNA-*Tdp*1 CL187 cells were treated with different concentrations of PTE and RE for 48 h, followed by flow cytometry. Total apoptosis was quantified by flow cytometry. *P < 0.05, **P < 0.01, and ***P < 0.001 vs. control. #P < 0.05, ##P < 0.01 and ###P < 0.001 vs. RE treatment group. ns, not significant. Data are mean ± SD, n=3.

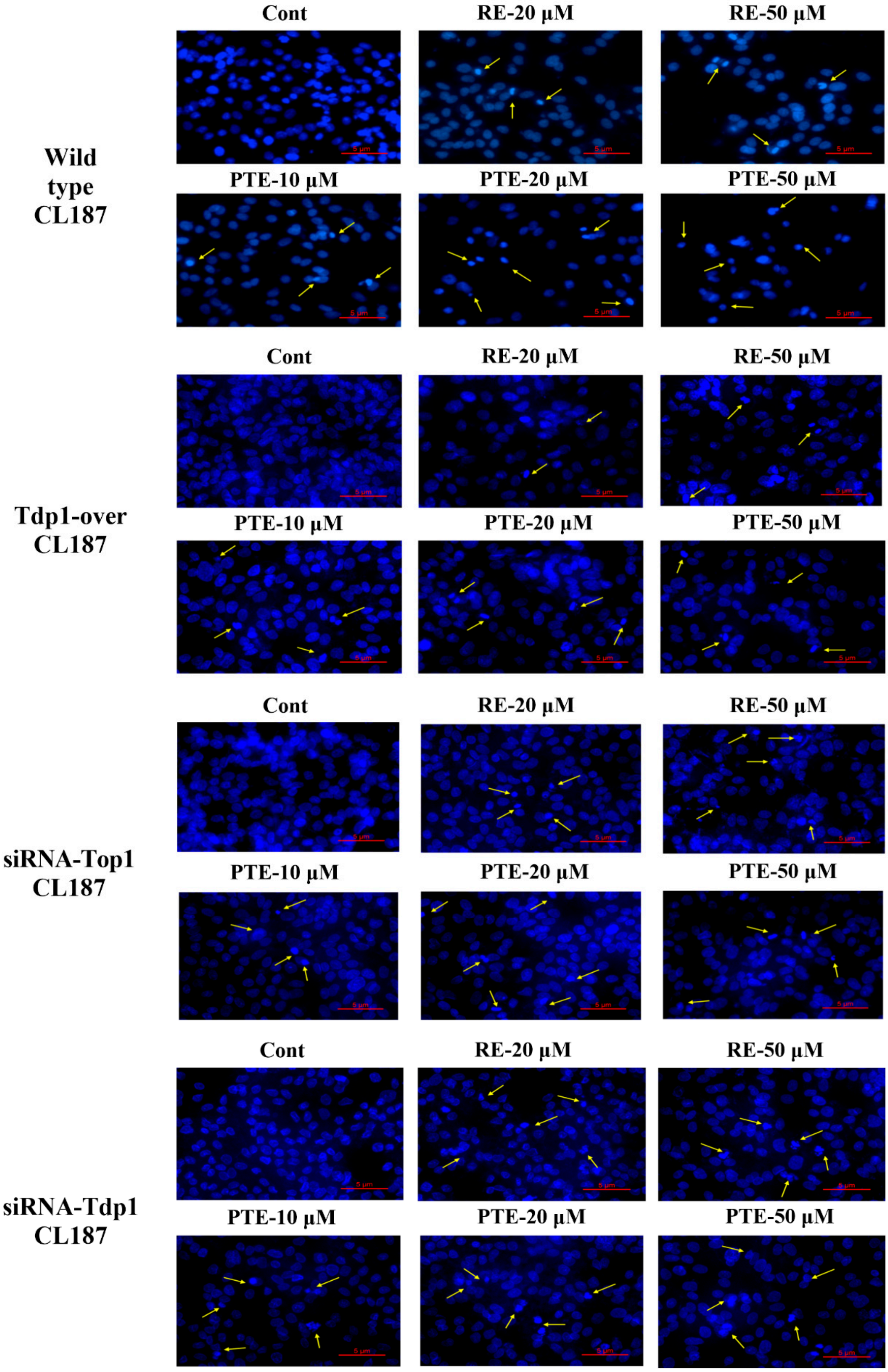

Figure S6. *Tdp*1-over CL187 cells, siRNA-*Top*1 CL187 cells and siRNA-*Tdp*1 CL187 cells were treated with different concentrations of PTE and RE for 48h, and stained cells were observed under a multimode microplate detection and cell imaging system (×400). the cells with condensed chromatin and shrunken nuclei were counted as apoptotic cells, marked with yellow arrows

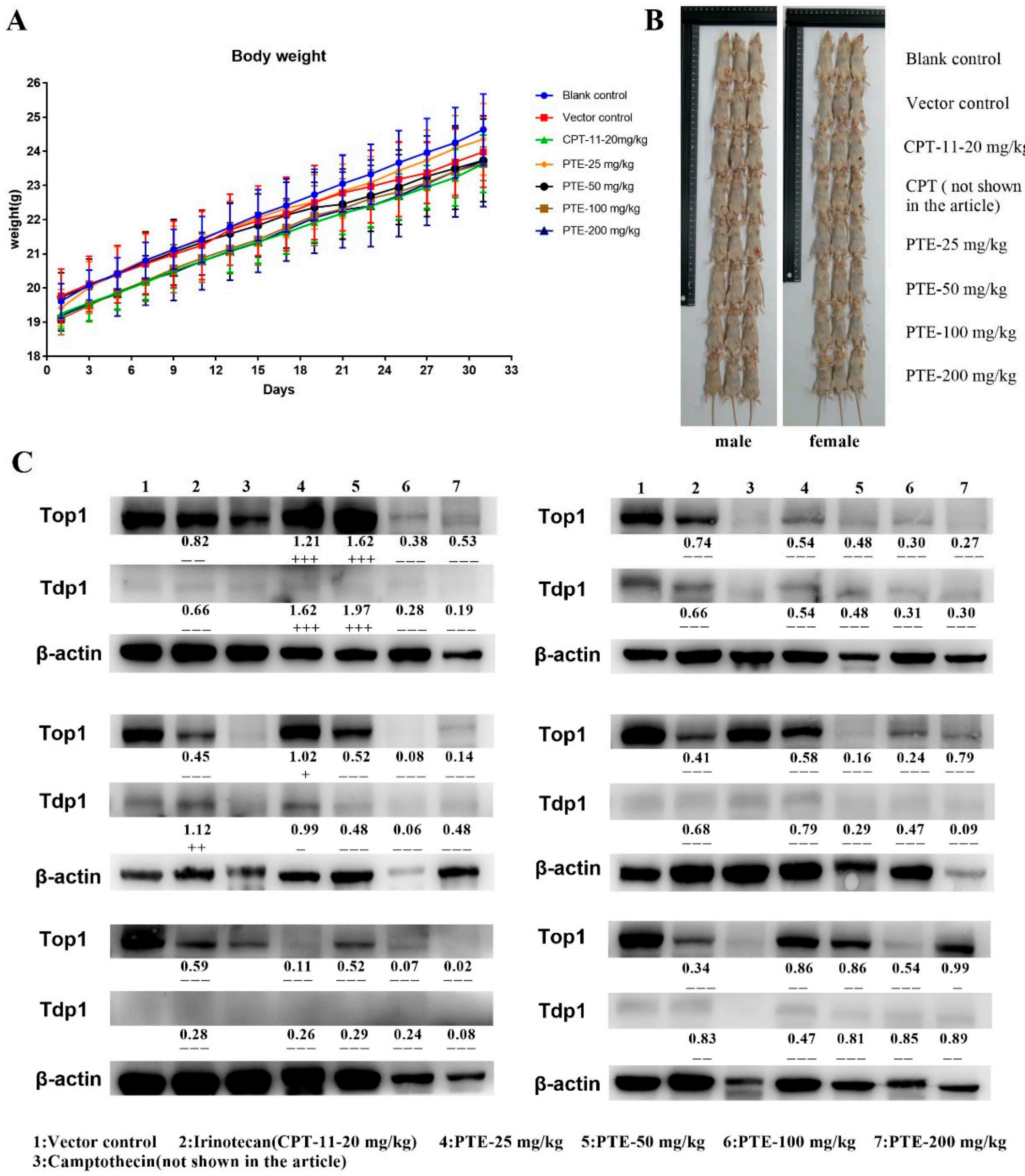

Figure S7. (A) Body weight of the subcutaneous CL187 xenograft nude mice. (B) Photograph of the mice in last day. Data are mean ± SD, n=6. (C) WB probing for Top1 and Tdp1 in tumor tissues. Number was relative Top1/Tdp1 level with control, loading was normalized based on the level of the internal control, β-actin. Compared with the control group,"+" or "-" was denoted when the change of gray value was between 0 and 10%, "++" or "--" was denoted when the change of gray value was between 10%-20%, "+++" or "---" was denoted when the change of gray value exceeded 20%.

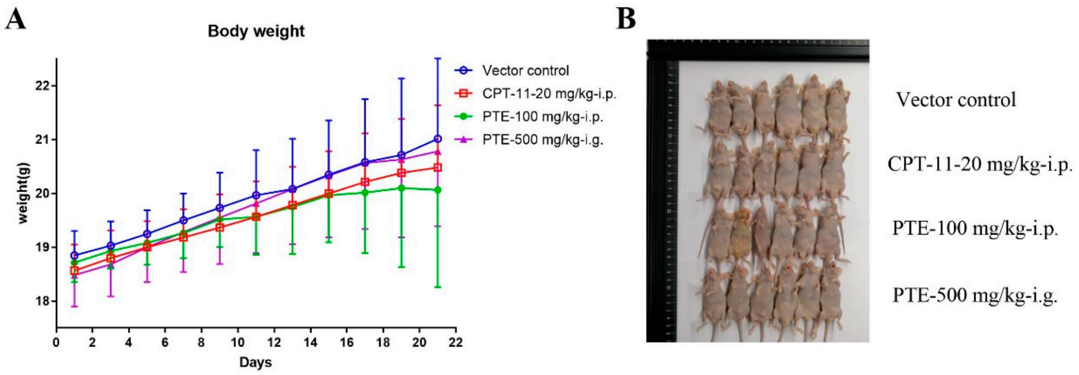

Figure S8. (A) Body weight of orthotopic CL187 xenograft nude mice. (B) Photograph of the mice in last day. Data are mean ± SD, n=6.
